# Species-specific cleavage of cGAS by picornavirus protease 3C disrupts mitochondria DNA-mediated immune sensing

**DOI:** 10.1371/journal.ppat.1011641

**Published:** 2023-09-14

**Authors:** Ya Yan, Lei Wu, Ye Yuan, Haiwei Wang, Hongyan Yin, Minjie Li, Lvye Chai, Ruiying Liang, Yanjie Liu, Dongming Zhao, Junji Xing, Pingwei Li, Xin Li

**Affiliations:** 1 National Key Laboratory of Veterinary Public Health and Safety, College of Veterinary Medicine, China Agricultural University, Beijing, China; 2 Key Laboratory of Animal Epidemiology of the Ministry of Agriculture and Rural Affairs, College of Veterinary Medicine, China Agricultural University, Beijing, China; 3 State Key Laboratory of Animal Disease Control, Harbin Veterinary Research Institute, Chinese Academy of Agricultural Sciences, Harbin, China; 4 Institute of Animal Sciences, Chinese Academy of Agricultural Sciences, Beijing, China; 5 College of Animal Sciences, Shanxi Agricultural University, Jinzhong, China; 6 Department of Surgery and Immunobiology and Transplant Science Center, Houston Methodist Research Institute, Houston Methodist, Houston, Texas, United States of America; 7 Department of Cardiovascular Sciences, Houston Methodist Research Institute, Houston Methodist, Houston, Texas, United States of America; 8 Department of Biochemistry and Biophysics, Texas A&M University, College Station, Texas, United States of America; Florida State University, UNITED STATES

## Abstract

RNA viruses cause numerous infectious diseases in humans and animals. The crosstalk between RNA viruses and the innate DNA sensing pathways attracts increasing attention. Recent studies showed that the cGAS-STING pathway plays an important role in restricting RNA viruses via mitochondria DNA (mtDNA) mediated activation. However, the mechanisms of cGAS mediated innate immune evasion by RNA viruses remain unknown. Here, we report that seneca valley virus (SVV) protease 3C disrupts mtDNA mediated innate immune sensing by cleaving porcine cGAS (pcGAS) in a species-specific manner. Mechanistically, a W/Q motif within the N-terminal domain of pcGAS is a unique cleavage site recognized by SVV 3C. Three conserved catalytic residues of SVV 3C cooperatively contribute to the cleavage of pcGAS, but not human cGAS (hcGAS) or mouse cGAS (mcGAS). Additionally, upon SVV infection and poly(dA:dT) transfection, pcGAS and SVV 3C colocalizes in the cells. Furthermore, SVV 3C disrupts pcGAS-mediated DNA binding, cGAMP synthesis and interferon induction by specifically cleaving pcGAS. This work uncovers a novel mechanism by which the viral protease cleaves the DNA sensor cGAS to evade innate immune response, suggesting a new antiviral approach against picornaviruses.

## Author summary

While the effect of cGAS on counteracting DNA viruses has been extensively studied, how cGAS restricts RNA viruses and how RNA viruses combat cGAS-mediated immune surveillance are largely unknown. Here, we show that picornavirus, seneca valley virus (SVV), infection causes the leakage of mitochondrial DNA into cytosol for cGAS activation, while SVV 3C protease cleaves porcine cGAS (pcGAS) in a species-specific manner to disrupt pcGAS-mediated DNA sensing, cGAMP synthesis and interferon induction. Importantly, we identify a W/Q motif within the N-terminal domain of pcGAS, which is unique among other species for SVV 3C cleavage. Three conserved catalytic residues of SVV 3C cooperatively contribute to the cleavage of pcGAS. These findings unravel a novel strategy for viral evasion of cGAS-mediated innate restriction.

## Introduction

Cyclic GMP-AMP synthase (cGAS) is an essential DNA sensor, which binds to viral dsDNA or mitochondria DNA (mtDNA), catalyzing the synthesis of 2’3’-cGAMP (cGAMP) [1–4]. This asymmetric second messenger binds and activates stimulator of interferon genes (STING), for subsequent induction of type I interferons (IFN-I) and IFN-stimulated genes (ISGs) to restrict viral replication [5,6]. Meanwhile, DNA viruses have evolved multiple strategies to evade cGAS-mediated host innate immune response. For example, Kaposi’s sarcoma-associated herpesvirus (KSHV) ORF52 interacts with both DNA and cGAS to inhibit the enzymatic activity of cGAS and suppress 2’,3’-cGAMP induced IFN-I production [7]. Human cytomegalovirus (HCMV) UL31 directly interacts with cGAS, causing the disassociation of DNA from cGAS and thus reduces cGAMP production [8]. Vaccinia virus (VACV) encoded F17 competitively sequesters Raptor and Rictor to dysregulate mTOR to increase cGAS degradation and suppressed DNA sensing [9]. Another study shows that Herpes simplex virus type 1 (HSV-1) UL37 deamidates post-translational modification of cGAS to abolish cGAMP synthesis in a species-specific manner [10]. In addition, porcine circovirus type 2 (PCV2) infection causes the phosphorylation of porcine cGAS via gC1qR-mediated phosphatidylinositol-3-kinase (PI3K)/AKT signaling pathway to abolish cGAS catalytic activity and promotes ubiquitination of cGAS for degradation [11].

Besides the major function of antagonizing for DNA viruses, recent studies show cGAS is an important restriction factor for RNA viruses [12,13]. Although cGAS also binds to RNA and RNA-DNA hybrids, RNA binding does not activate cGAS to synthesize cGAMP [14]. The molecular mechanism of RNA virus restriction mediated by cGAS remains largely unexplored. Two groups reported that Dengue virus (DENV) infection resulted in the mtDNA release into the cytosol, which activates cGAS to restrict viral replication [15,16]. A growing number of studies showed that diverse RNA viruses including zika virus (ZIKA), chikungunya virus (CHIKV), influenza A virus (IAV), encephalomyocarditis virus (EMCV) and porcine reproductive and respiratory syndrome virus (PRRSV) could activate cGAS by triggering the releases of mtDNA [17–20].

Recently, it was reported that picornaviruses, such as enterovirus 71 (EV-A71), seneca valley virus (SVV) and foot-and-mouth disease virus (FMDV), could activate cGAS by triggering mitochondria damage and mtDNA release *in vitro* and *vivo* [21]. In addition, SVV 2C protein was shown to be responsible for cGAS reduction via autophagy pathway. The non-structural protein 3C protease encoded by SVV cleaves viral polyproteins to assemble viral replication complex during virus replication. Furthermore, SVV 3C protease was shown to not only cleave gasdermin D to induce caspase-1 mediated pyroptosis [22] but also cleave mitochondrial antiviral signaling (MAVS), Toll/interleukin 1 (IL-1) receptor domain-containing adaptor inducing IFN-β (TRIF), and TRAF family member-associated NF-κB activator (TANK) to antagonize antiviral innate immune responses [23]. There are a number of studies showing different kinds of modifications of cGAS by viral and host proteins for regulating cGAS activation. To date, there is very few evidence to show the direct cleavage of cGAS by viral or host proteins for suppressing cGAS activation.

In this study, we employed multiple biochemical methods and cell-based assays, to show that pcGAS was directly cleaved by SVV 3C protease in species-specific manner. The 3C proteins from other picornaviruses including EV-A71, hepatitis A virus (HAV), coxsackievirus B3 (CVB3), poliovirus (PV) and human rhinovirus (HRV) did not cause the cleavage of human cGAS (hcGAS). Mechanistically, a W/Q motif in pcGAS that is not conserved in cGAS from other species is cleaved by SVV 3C. Three conserved catalytic residues (H48, D84 and C160) in SVV 3C cooperatively contributed to pcGAS cleavage. In cells, SVV WT 3C colocalized with pcGAS and the co-localization was enhanced upon poly(dA:dT) stimulation and SVV infection. Furthermore, cleavage of pcGAS NT significantly dampened pcGAS binding to dsDNA and cGAMP production. Collectively, our data unveils a novel strategy wherein a viral protease directly cleaves the DNA sensor cGAS and thus limits mtDNA sensing to antagonize innate immune signaling.

## Results

### The activation of cGAS-STING pathway restricts SVV replication

cGAS is activated by dsDNA to synthesize cGAMP that binds to STING to induce the production of IFN-β. Several recent studies showed that cGAS also robustly restricts RNA viruses [13,19,21]. To determine whether SVV infection compromises antiviral responses via the cGAS-STING pathway, we stimulated swine testis (ST) cells with different doses of poly(dA:dT) and 2’,3’-cGAMP for 6 h and then infected the cells with SVV (MOI = 0.1) for 0, 6, 12 and 24 h to assess viral replication via quantifications of SVV 3C and VP1 genes by qRT-PCR assay. In comparison to unstimulated cells, the gene copies of SVV 3C and VP1 were significantly decreased in a dose dependent manner in ST cells transfected with poly (dA:dT) (100, 250 and 500 ng) (S1A and S1B Fig). Since cGAMP is the ligand for STING to activate downstream antiviral innate immune signaling, the copy numbers of SVV 3C and VP1 were significantly reduced in ST cells transfected with cGAMP (200, 500 and 1000 ng) compared with unstimulated group in a dose-dependent manner (S1C and S1D Fig).

**Fig 1:**
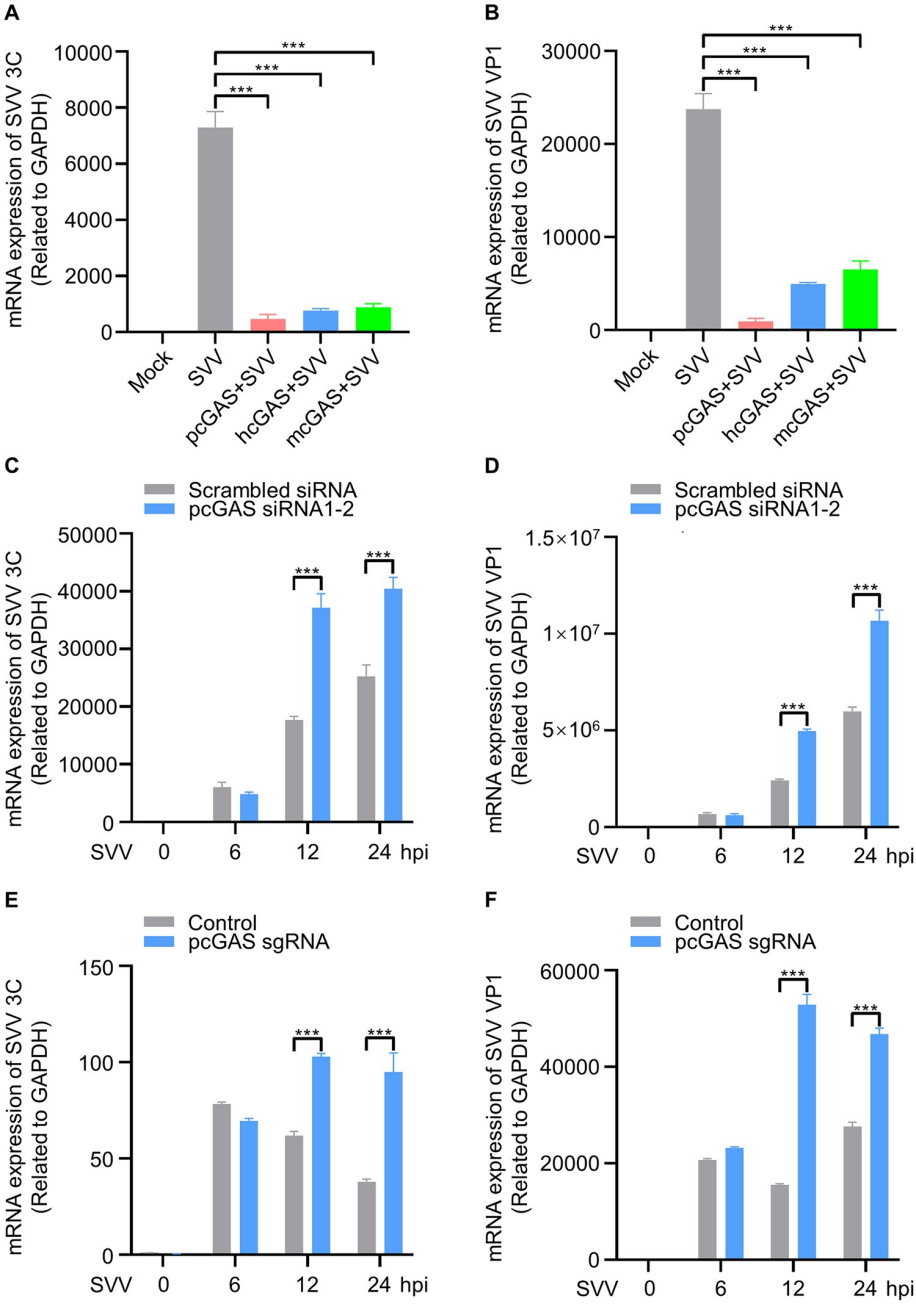
The activation of cGAS-STING pathway restricts SVV replication. **A-B**, The RT-qPCR analysis of SVV 3C (A) and VP1 (B) mRNA expression (related to GAPDH) in ST cells mock-transfected or transfected with pcGAS/hcGAS/mcGAS for 24 h, followed by infection with SVV (MOI = 0.1) for 12 h. **C-D**, The RT-qPCR analysis of SVV 3C (**C**) and VP1 (**D**) mRNA expression (related to GAPDH) in PK-15 cells transfected with scrambled siRNA or siRNA targeting *pcGA*S for 36 h, followed by infection with SVV (MOI = 0.1) for another 6, 12 and 24 h. **E-F**, The RT-qPCR analysis of SVV 3C (**E**) and VP1 (**F**) mRNA expression (related to GAPDH) in ST cells with scrambled or sgRNA targeting *pcGAS*, followed by infection with SVV (MOI = 0.1) for another 6, 12 and 24 h. Results are representative of three biological replicates. Means ± SD are shown in **A-F** (n = 3). Two-tailed unpaired *t*-test was used for the statistical analysis, ***P < 0.001.

The impact of cGAS on SVV replication in porcine cells was further tested by overexpression of pcGAS, hcGAS, and mcGAS in ST cells and then infected with SVV (MOI = 0.1) for 12 h. The results indicated that overexpression of pcGAS, hcGAS, and mcGAS could significantly inhibit SVV replication (Fig 1A and 1B). To obtain a more physiological relevant understanding of the role of cGAS-STING pathway in SVV infection, we used RNAi to knockdown of *pcGAS* expression in porcine kidney-15 (PK-15) cells and knockout of *pcGAS* in ST cells via CRISPR/Cas9. Knockdown of *pcGAS* in PK-15 cells significantly increased 3C and VP1 mRNA expression at 12 and 24 h post infection (MOI = 0.1) compared to scramble during SVV replication (Fig 1C and 1D). In addition, we also generated pcGAS knockout (KO) ST cells by CRISPR/Cas9 technology. The data indicated that the copy numbers of SVV 3C and VP1 mRNA in pcGAS KO cells were significantly higher than pcGAS sufficient cells at 12 and 24 h after infection of SVV (MOI = 0.1) (Fig 1E and 1F). All these data suggested pcGAS restricts SVV replication in porcine cells. Collectively, these results suggest that cGAS-STING pathway activation directly restricts SVV replication.

### SVV infection induces the release of mtDNA into the cytosol

cGAS senses viral DNA and cellular DNA that is abnormally present within the cytosol to activate downstream innate immune responses. Since the viral genome of SVV is RNA, we hypothesize that SVV infection likely induces damage or dysfunction of mitochondria and causes the release of mtDNA into the cytosol, which can be recognized by pcGAS. To test this hypothesis, we detected the presence of mtDNA in the cytosol during SVV infection. We infected HEK-293T cells with SVV (MOI = 10) for 6, 9, 12, 15 and 18 h and then lysed the cells followed by isolation of the cytosol at each time point. Quantitative PCR (qPCR) was used to quantify the abundance of mtDNA, which showed dramatic increase of mtDNA after SVV infection (S2A Fig). To further determine whether this happens in porcine cells, we infected PK-15 cells with SVV (MOI = 10) for 6, 9, 12, 15 and 18 h and then lysed the cells followed by isolation of the cytosolic fraction at each time point. The relative abundance of mtDNA significantly increased at 12, 15 and 18 h post infection in PK-15 cells (S2B Fig). Taken together, these data suggest that SVV infection causes leakage of mtDNA into cytosol that activates cGAS.

### SVV protease 3C cleaves porcine cGAS but not human or mouse cGAS

**Fig 2:**
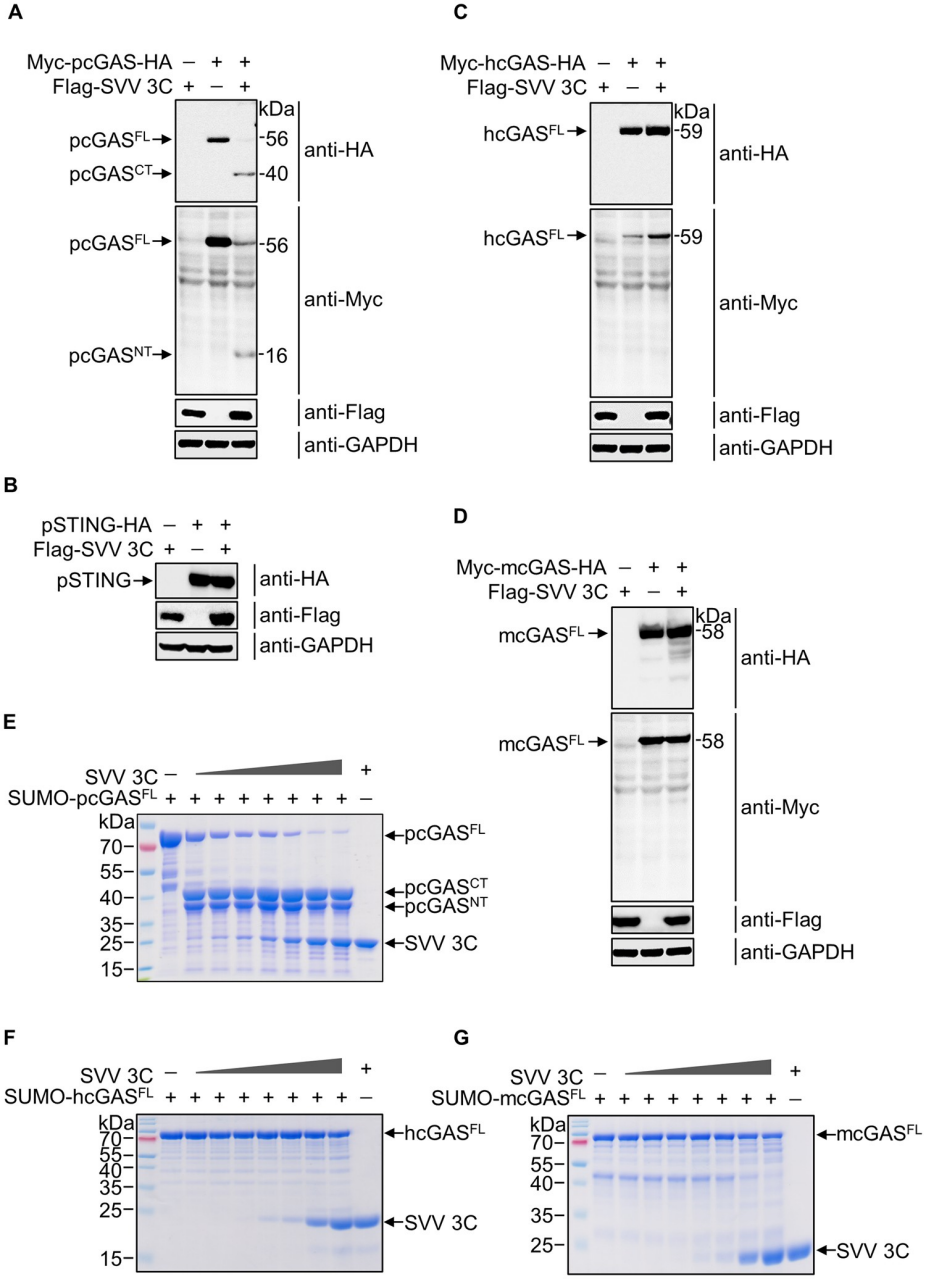
SVV protease 3C cleaves porcine cGAS but not human or mouse cGAS. **A-D**, Western blot analysis of cGAS or STING cleavage and SVV 3C expression in HEK-293T cells transfected with a plasmid encoding SVV 3C, together with a plasmid encoding pcGAS (**A**), pSTING (**B**), hcGAS (**C**) or mcGAS (**D**) for 24 h using anti-Myc, anti-HA and anti-Flag antibodies. **E-G**, SDS-PAGE analysis of *in vitro* cleavage of pcGAS (**E**), hcGAS (**F**) or mcGAS (**G**) in a 25-μl reaction containing 20 μg SUMO-pcGAS^FL^ (**E**), SUMO-hcGAS^FL^ (**F**), or SUMO-mcGAS^FL^ (**G**) recombinant protein with different amounts of purified recombinant protein SVV 3C (0.01, 0.05, 0.1, 0.5, 1, 5, 10 μg) for 2 h at 37°C.

Picornavirus protease 3C has been reported to cleave many RNA sensors and adaptors, such as MDA-5, MAVS, TRIF [23]. Considering the pivotal role of the cGAS-STING pathway in restricting SVV replication, we tested whether picornavirus protease 3C directly cleaves cGAS or STING to evade innate immune sensing. We first co-expressed a FLAG-tagged SVV 3C along with Myc-pcGAS-HA and HA-tagged porcine STING (pSTING) in HEK-293T cells, respectively. We observed two clear bands of pcGAS (~40 kDa and 16 kDa) when co-expressing SVV 3C and pcGAS (Fig 2A). This result confirmed SVV 3C was capable of cleaving pcGAS. Because DENV and Zika NS2B3 cleaved human STING [24,25]. We sought to test whether SVV 3C also cleave pSTING. Interestingly, we did not observe the characteristic cleavage products when we co-expressed SVV 3C and pSTING, suggesting that pSTING is not the target for SVV 3C (Fig 2B). It is reported that deamidation of a single asparagine in cGAS determines species-specific inactivation by HSV-1 [8]. To determine if cGAS cleavage by SVV 3C is specific to pcGAS, we co-expressed SVV 3C with hcGAS and mcGAS, respectively. We did not detect the cleavage of hcGAS and mcGAS by SVV 3C (Fig 2C and 2D). To test if the cleavage of pcGAS by SVV 3C is direct, we purified full-length of porcine cGAS (pcGAS^FL^), human cGAS (hcGAS^FL^) and mouse cGAS (mcGAS^FL^) proteins with an N-terminal His_6_-SUMO-FLAG and a C-Terminal HA tag (S3A, S3B and S3C Fig). Considering the NT of full-length cGAS is very flexible and susceptible to degradation in solution, we kept the SUMO-tag on N terminus in cGAS^FL^ for stabilization. We also purified His_6_-SUMO-pcGAS catalytic domain (SUMO-pcGAS^CAT^) for cleavage assay (S3D Fig). Furthermore, we purified SVV 3C and EV-A71 3C proteins for the cleavage assays (S3E and S3F Fig). Based on the conditions used for gasdermin D cleavage, the cleavage assay buffer contains 50 mM HEPES PH7.5, 3 mM EDTA, 150 mM NaCl, 0.005% Tween20 and 10 mM DTT [26]. We next incubated SVV 3C with pcGAS^FL^, hcGAS^FL^ and mcGAS^FL^ at 37°C. After 2 hours, the mixtures were analyzed by SDS-PAGE. The pcGAS^FL^ band gradually disappeared depending on the different amounts of SVV 3C used. Two new bands with a molecular mass of ~40 kDa (CT) and NT including SUMO tag (Fig 2E) appeared, indicating that pcGAS^FL^ was processed into two major fragments. In contrast, hcGAS^FL^ and mcGAS^FL^ were intact when incubated with SVV 3C (Fig 2F and 2G). To explore if other picornavirus 3C could cleave cGAS, we co-expressed a FLAG-tagged EV-A71 3C along with pcGAS, hcGAS and mcGAS in HEK-293T cells. Surprisingly, we did not detect the cleavage of pcGAS, hcGAS and mcGAS by EV-A71 3C (S4A, S4B and S4C Fig), which is consistent with the recently published data [21], wherein EV-A71 infection did not affect the expression of hcGAS in both Hela and HT-29 cells. Furthermore, we showed that pcGAS^CAT^, hcGAS^FL^ and mcGAS^CAT^ remained intact when incubated with EV-A71 3C (S4D, S4E and S4F Fig).

*Picornaviridae* is an important and large family of RNA viruses, including SVV, EV-A71, HAV, CVB3, PV and HRV. To test if other picornaviruses protease 3C also cleave cGAS, we synthetized HAV, CVB3, PV and HRV 3C genes. Since all these viruses are susceptible to human and we tested if they could cleave hcGAS in HEK-293T cells. Similarly, HAV, CVB3, PV and HRV protease 3C did not cleave hcGAS in the transfected cells (S5A, S5B, S5C and S5D Fig). Taken together, our data suggest that SVV 3C directly cleaves pcGAS in species-specific manner.

### SVV infection causes the cleavage of pcGAS within its N-terminal domain

**Fig 3:**
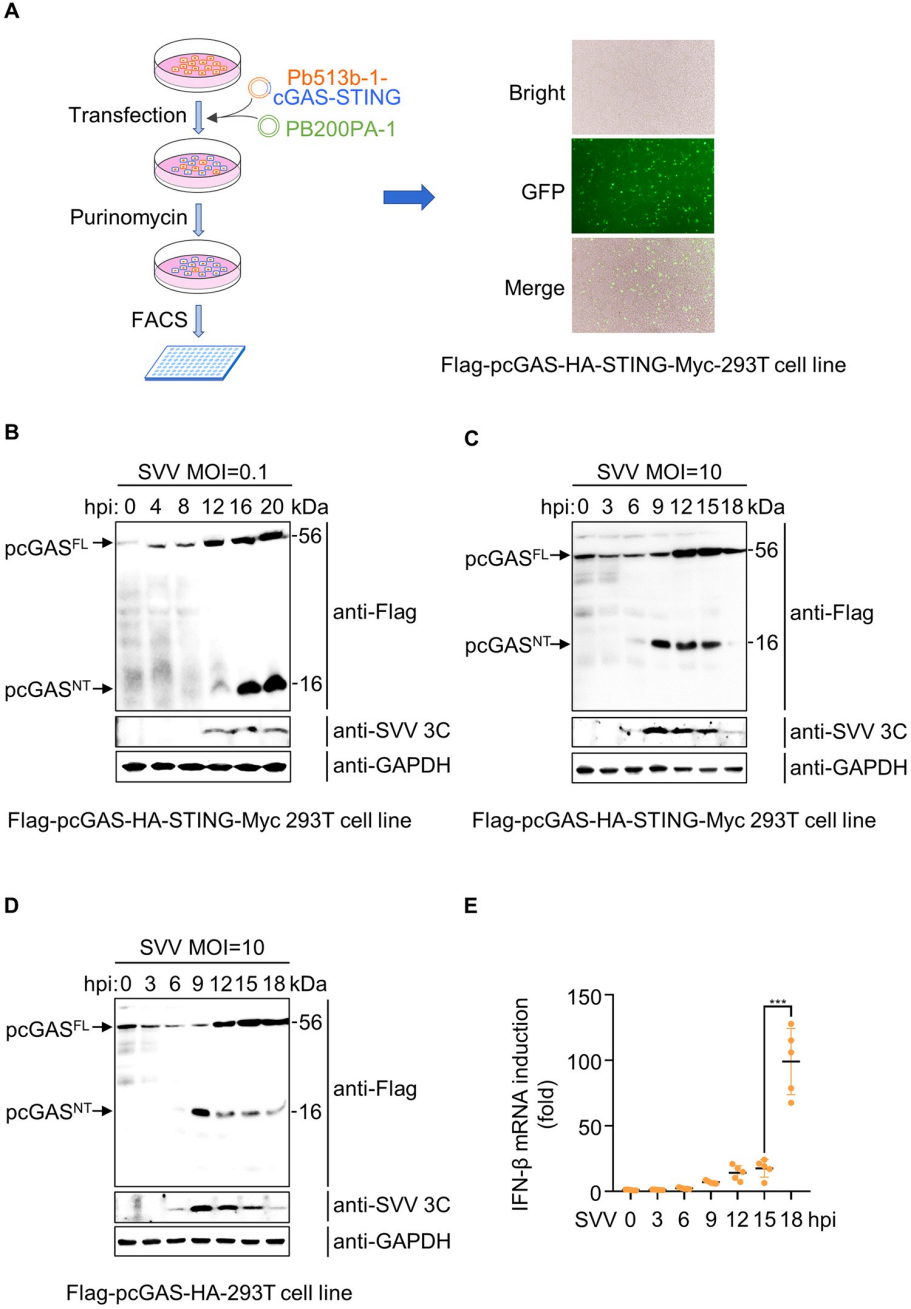
SVV infection causes the cleavage of pcGAS within its N-terminal domain. **A**, Construction of HEK-293T cell line stably expressing pcGAS alone or both pcGAS and pSTING using PiggyBac Transposon system by transfecting HEK-293T cells with PB200PA-1 plasmid, together with cGAS-STING or cGAS expressing plasmid (Pb513b-1-cGAS-STING or Pb513b-1-cGAS plasmid), followed by purinomycin screening and flow separation. **B,** Western blot analysis of pcGAS cleavage in HEK-293T cell line stably expressing both pcGAS and pSTING mock or infected with SVV at MOI of 0.1 for 0, 4, 8, 12, 16 or 20 h. **C**, **D**, Western blot analysis of pcGAS cleavage in HEK-293T cell line stably expressing both pcGAS and pSTING (**C**) or pcGAS only (**D**) mock or infected with SVV at MOI of 10 for 0, 3, 6, 9, 12,15 or 18 h. **E**, The RT-qPCR analysis of IFN-β mRNA in HEK-293T cell line stably expressing both pcGAS and pSTING mock or infected with SVV at MOI of 10 for 0, 3, 6, 9, 12,15 or 18 h. Results are representative of five biological replicates. Means ± SD are shown in **E** (n = 5). Two-tailed unpaired *t*-test was used for the statistical analysis, ***P < 0.001.

We confirmed that SVV 3C could specifically cleave pcGAS *in vitro* and in HEK-293T cells. To provide a physiological context, we investigated whether pcGAS could be cleaved in a multiplicity of infection (MOI)-dependent manner. Due to lack of pcGAS endogenous antibody, we constructed a cell line stably expressing pcGAS-pSTING in HEK-293T cells using PiggyBac Transposon system (Fig 3A, left). We observed the fluorescence intensity of GFP in the cell line stably expressing pcGAS-pSTING by fluorescence microscopy (Fig 3A, right). To determine whether pcGAS was cleaved upon infection, we challenged HEK-293T cells stably expressing pcGAS-pSTING with SVV (MOI = 0.1 and 10) and collected the cells at different time points post infection. pcGAS NT was detected by the anti-FLAG antibody. Consistent with the data shown in Fig 2, western blot showed that an obvious band on the bottom of the gel at 16 and 20 h post infection (MOI = 0.1) and the molecular mass of ~16 kDa indicating the product is pcGAS NT (Fig 3B). Unfortunately, we did not detect pcGAS CT with the anti-HA tag antibody. We speculated that the poor recognition ability of anti-HA tag antibody may lead to the inability to detect pcGAS CT with lower sensitivity. To confirm these results, we repeated this experiment at higher infection intensity (MOI = 10). As expected, we observed the same pcGAS NT band at 9, 12 and 15 h post infection, when the NT product appeared earlier than infection (MOI = 0.1) (Fig 3C). To further confirm pcGAS but not pSTING was cleaved by SVV 3C protease, we also generated HEK-293T cell line stably expressing pcGAS without expressing pSTING via PiggyBac Transposon system. This cell line was challenged with SVV (MOI = 10). Consistent with the data obtained using HEK-293T cell line stably co-expressing pcGAS-pSTING, pcGAS was cleaved at 9, 12, 15 and 18 h post infection (Fig 3D). Subsequently, we tested whether the endogenous hcGAS in Hela and RD cells could be cleaved upon picornavirus infection. Hela cells were infected with SVV (MOI = 10) or EV-A71(MOI = 10) and RD cells were infected with EV-A71(MOI = 10). Cytopathic effect (CPE) in Hela cells upon SVV infection showed clearly at 30 h post infection (S6A Fig, left). However, hcGAS was not cleaved after SVV infection in Hela cells (S6A Fig, right). In addition, a significant CPE in Hela or RD cells was observed at 12 h post infection (S6B and S6C Fig, left). Similarly, the expression level of hcGAS did not change at 6 and 12 h upon EV-A71 infection (S6B and S6C Fig, right). These results indicated that SVV and EV-A71 infection did not cause the cleavage of hcGAS on endogenous level. Because HEK-293T cell line stably expressing pcGAS-pSTING contains all the components for IFN-I induction, we tested IFN-β mRNA at different time points after SVV infection (MOI = 10). The expression of IFN-β gradually increased 9,12 and 15 h and significantly enhanced 18 h post infection (Fig 3E). These data suggested that IFN-β expression was suppressed at early time points because pcGAS was cleaved by SVV 3C. Taken together, these data demonstrate that pcGAS is cleaved within its N-terminal domain upon SVV infection.

### SVV 3C cleaves a unique W/Q motif within the NT of pcGAS

**Fig 4:**
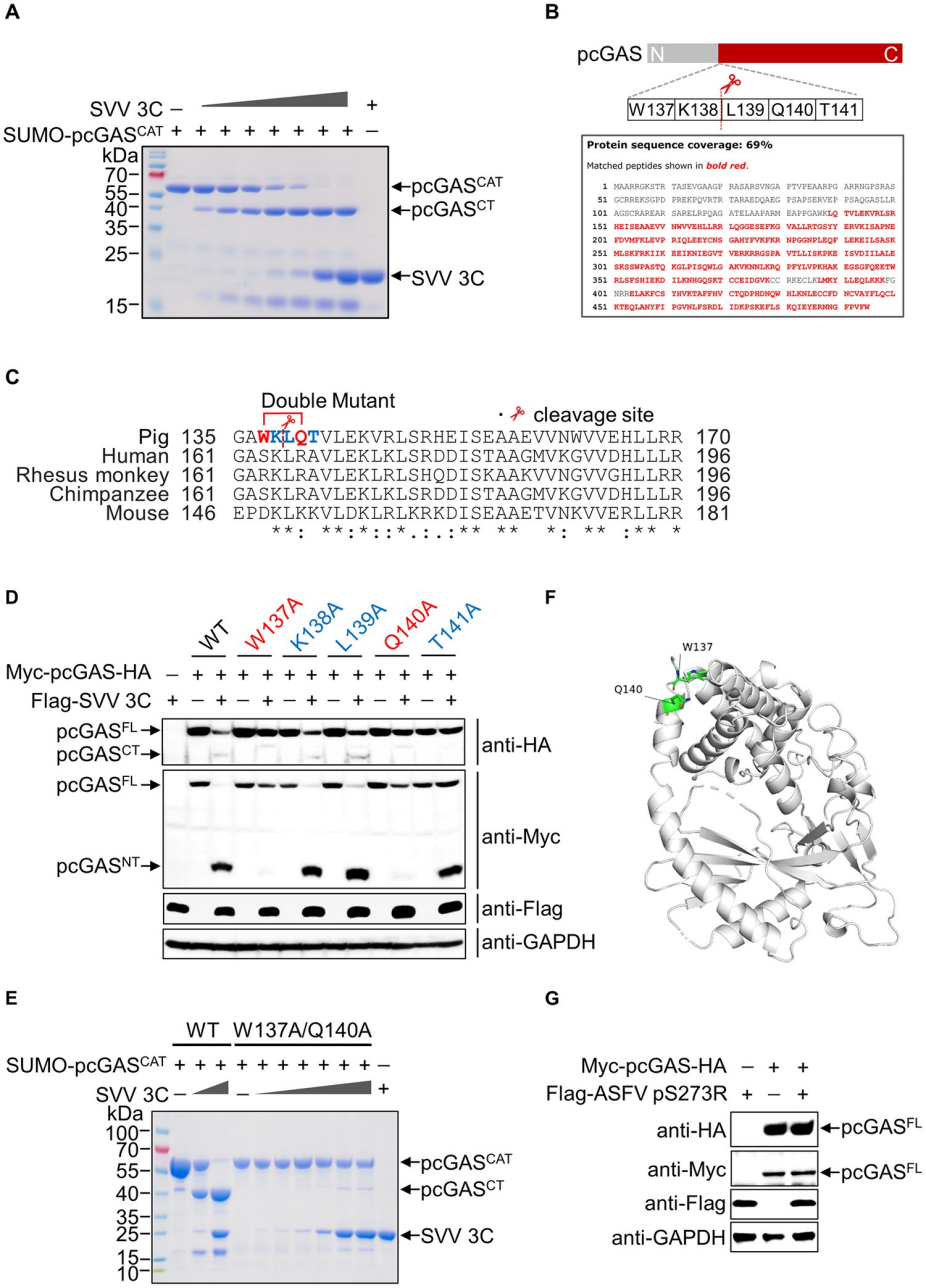
SVV 3C cleaves pcGAS NT with a unique W/Q motif. **A**, SDS-PAGE analysis of *in vitro* cleavage of pcGAS^CAT^ in a 25-μl reaction containing 20 μg SUMO-pcGAS^CAT^ recombinant protein with different amounts of purified recombinant protein SVV 3C (0.01, 0.05, 0.1, 0.5, 1, 5, 10 μg) for 2 h at 37°C. **B**, The cleavage site of pcGAS and protein sequence analysis of pcGAS^CT^ band cut for mass spectrometry identification. The sequence of pcGAS^CT^ is shown in red. **C**, Sequence alignment of cGAS from various primate species. **D**, Western blot analysis of pcGAS cleavage and SVV 3C expression in HEK-293T cells transfected with 1.5 μg SVV 3C plasmid and 2 μg wild-type or mutant pcGAS (pcGAS W137A, pcGAS K138A, pcGAS L139A, Q140A or T141A) expression plasmid for 24 h using anti-Myc, anti-HA and anti-Flag antibodies. **E**, SDS-PAGE analysis of *in vitro* cleavage of pcGAS^CAT^ in a 25-μl reaction containing 20 μg SUMO-WT pcGAS^CAT^ or SUMO-mutant pcGAS^CAT^ W137A/Q140A recombinant protein with different amounts of purified recombinant protein SVV 3C (0.01, 0.05, 0.1, 0.5, 1, 5, 10 μg) for 2 h at 37°C. **F**, The W137 and Q140 amino acids are displayed in the structure of pcGAS^CAT^ (PDB: 4JLX) using PyMOL analysis software. **G**, Western blot analysis of pcGAS cleavage and ASFV ps273R expression in HEK-293T cells transfected with 1.5 μg FLAG-ASFV pS273R plasmid and 2 μg wild-type Myc-pcGAS-HA plasmid for 24 h using anti-Myc, anti-HA and anti-Flag antibodies.

To determine the precise site of cleavage, we incubated SUMO-pcGAS^CAT^ with SVV 3C. The major cleavage products of ~40 kDa was excised from the SDS-PAGE gel and digested with trypsin (Fig 4A). The first identifiable peptide from the cleavage products starts from L139, which was located within pcGAS NT (Fig 4B). We aligned the sequences close to cleavage sites of pig, human, rhesus monkey, chimpanzee and mouse cGAS and observed that pcGAS has a unique W/Q motif that is not present in other species (Fig 4C). To determine the exact position of cleavage, we swapped W137, K138, L139, Q140 and T141 with alanine. Notably, mutations W137A and Q140A drastically reduced SVV 3C cleavage (Fig 4D). To further confirm this result, we purified W137A/Q140A double mutant SUMO-pcGAS^CAT^ protein (DM) (S7A Fig). We incubated SVV 3C with DM and we did not observe the characteristic cleavage pattern (Fig 4E). Next, we mapped these two key residues in the crystal structure of pcGAS^CAT^ (Fig 4F). SVV 3C is a chymotrypsin-like cysteine protease that is responsible for most of the cleavages during viral polyprotein processing [27]. To investigate if other type of cysteine protease also cleaves pcGAS, we constructed a FLAG-tagged pS273R, which is the SUMO-1 cysteine protease encoded from African swine fever virus (ASFV) [28]. While co-expression of ASFV FLAG-pS273R along with pcGAS, we did not detect the characteristic cleavage fragments, suggesting 3C specifically cleaves cGAS (Fig 4G). Collectively, these results demonstrate that the unique W/Q motif within pcGAS NT is specifically cleaved by SVV 3C.

### The protease activity of SVV 3C is required for pcGAS cleavage

**Fig 5:**
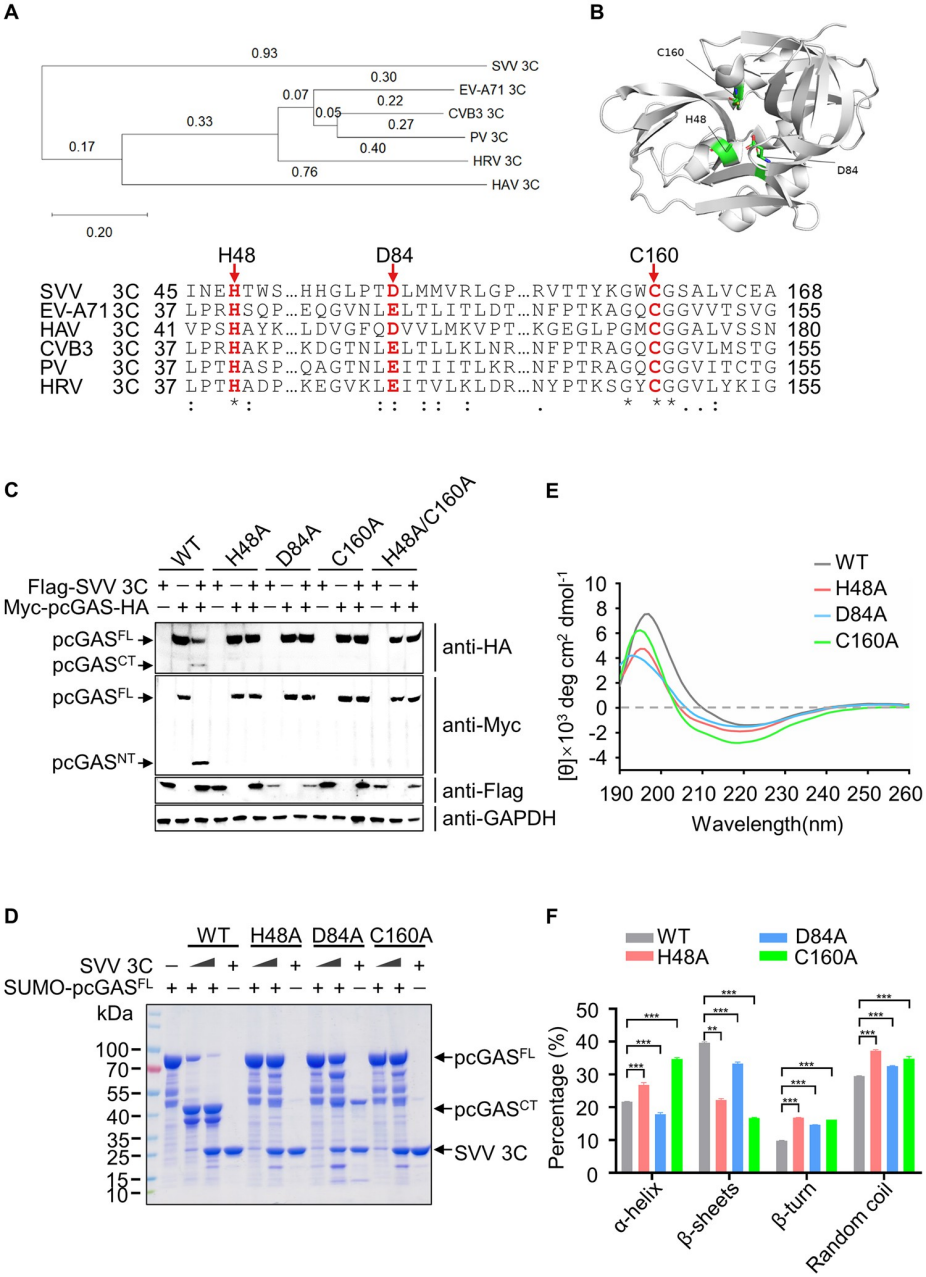
The protease activity of SVV 3C is required for pcGAS cleavage. **A**, The phylogeny and multiple sequence alignment analysis of 3C from picornaviruses. The key residues of 3C enzyme activity are displayed in red font in the sequence, including H48, D84 and C160. **B**, Visualization of the three catalytic residues in the structure of SVV 3C (PDB: 6L0T) through PyMOL. **C**, Western blot analysis of pcGAS cleavage and SVV 3C expression in HEK-293T cells transfected with 2 μg wild-type pcGAS and 1.5 μg wild-type or mutant SVV 3C (3C WT, 3C H48A, 3C D84A, 3C C160A or 3C H48A/C160A) expression plasmid for 24 h using anti-Myc, anti-HA and anti-Flag antibodies. **D**, SDS-PAGE analysis of *in vitro* cleavage of pcGAS in a 25-μl reaction containing 20 μg SUMO-pcGAS^FL^ recombinant protein with different amounts of purified recombinant protein wild-type or mutant SVV 3C (WT, H48A, H84A or C160A) for 2 h at 37°C. **E**, Circular dichroism spectrometry of purified recombinant protein SVV 3C and its mutants at 25°C in the buffer containing 10mM Tris-HCl. **F**, The data analysis of different secondary structures in SVV 3C and its mutants. Results are representative of three biological replicates. Two-tailed unpaired *t*-test was used for the statistical analysis, **P < 0.01, ***P < 0.001.

To further determine the properties of SVV protease 3C, we carried out phylogeny and multiple sequence alignment of picornaviruses 3C and drew a neighbor-Joining (NJ) tree by MEGA software. Obviously, the distance between SVV 3C and other picornaviruses are far away, suggesting a different role of SVV 3C (Fig 5A). We next aligned the amino acid sequences of SVV 3C with EV-A71, HAV, CVB3, PV and HRV 3C. Picornavirus protease 3C processes viral polyprotein to assemble the mature virus particle. The conserved catalytic residues of SVV 3C contain H48, D84 and C160 [23,29,30] (Fig 5A). The crystal structure of SVV 3C showed that it adopts a chymotrypsin like fold similar to other picornaviral 3C [31]. We mapped these conserved catalytic residues in the structure (Fig 5B). To test the functional relevance of these critical residues in SVV 3C, we constructed point mutations by swapping H48, D84, C160 and H48/C160 for alanine. We next co-expressed SVV 3C WT and its mutants with pcGAS^FL^. As a positive control, pcGAS^FL^ was cleaved by WT 3C, however, we did not detect the characteristic cleavage product with the three 3C mutants (Fig 5C). Furthermore, we also purified three SVV 3C mutant proteins and conducted an activity assay (S7B, S7C and S7D Fig). As expected, incubation of pcGAS^FL^ with the three catalytically inactive mutants did not result in the proteolytic cleavage of pcGAS^FL^ (Fig 5D).

To probe whether the catalytically inactive mutants affect the structure of the SVV 3C protein, we further used CD spectra to investigate the secondary structures in purified SVV 3C and its catalytic mutants (H48A, D84A and C160A). The results indicated that the secondary structure of the catalytically inactive mutants showed significant changes compared with WT, as there was an obvious shift of α-helix (192, 208, 222 nm), β-sheets (195, 216 nm), β-turn (205 nm), Random coil (200, 212 nm) signature peaks (Fig 5E). To investigate the exact content of SVV 3C and its mutants, the CD spectra were deconvoluted via CDNN software. In comparison with WT, the α-helix content in H48A and C160A increased, but D84A decreased. However, the β-sheets content in three mutants decreased (Fig 5F). It is reported that SVV 3C WT has an unusual structure of two neighboring β-turn, which is identified to affect substrate recognition [31]. The β-turn content in three mutants all significantly increased, probably leading to the changes of substrate cleavage activity (Fig 5F). Collectively, these data suggest that the mutants (H48, D84 and C160) induce secondary structural changes compared with WT.

To further confirm the stability of the proteins, Far-UV CD spectroscopy (190–260 nm) was used to measure the stability of the SVV 3C and its mutants at different temperatures. The composition of α-helix and β-sheets in SVV 3C WT and its mutant proteins were consistent at 25°C, 35°C and 45°C until a significant change at 55°C (S8 Fig). These results suggested that SVV 3C and its mutant proteins could maintain the original structure in thermodynamic stability. Taken together, these data demonstrate that the three conserved catalytic residues in SVV 3C are critical for pcGAS cleavage.

### SVV 3C co-localizes with pcGAS upon DNA stimulation or SVV infection

**Fig 6:**
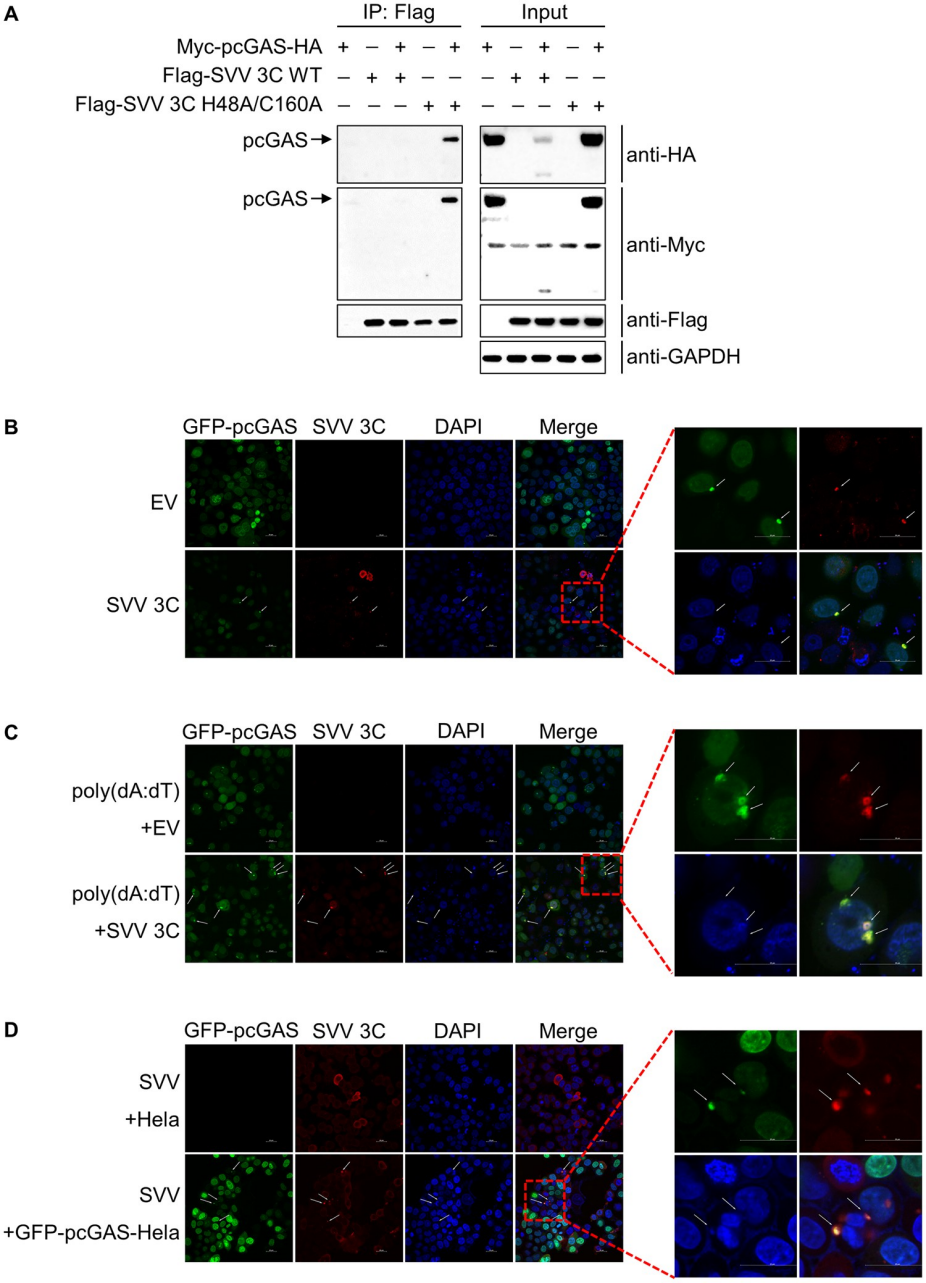
SVV 3C co-localizes with pcGAS upon DNA transfection or SVV infection. **A**, Co-IP assay for assessment of the interactions between pcGAS and 3C proteins in HEK-293T cells. After 24 h post-transfection of plasmids expressing pcGAS and 3C, the cells were lysed and the supernatants were immunoprecipitated with mouse anti-FLAG MAb. The immunoprecipitated complex was analyzed by immunoblotting with the indicated antibodies. **B**, The confocal microscopy analysis of colocalization between pcGAS and SVV 3C in Hela cells stably expressing pcGAS (GFP-pcGAS-Hela) transfected with empty vector or plasmid expressing wild-type 3C by confocal microscopy. Nuclei were stained with DAPI (blue). Green fluorescence indicated pcGAS, and red fluorescence indicated 3C. The arrows referred to the co-localization of pcGAS and 3C. **C**, **D**, The confocal microscopy analysis of colocalization between pcGAS and SVV 3C in GFP-pcGAS-Hela cells transfected with empty vector or plasmid expressing wild-type 3C followed by poly(dA:dT) stimulation (**C**) or SVV infection at MOI of 10 (**D**) by confocal microscopy. Arrows marked co-localization of SVV 3C and pcGAS. Scale bars, 20 μm.

To address the interaction between SVV 3C and pcGAS, HEK-293T cells were transfected with pcGAS and either 3C WT or 3C H48A/C160A and co-immunoprecipitation studies were performed. The results indicated that whereas 3C H48A/C160A and pcGAS co-immunoprecipitated, 3C WT and pcGAS did not. We speculate the reason is that the SVV 3C WT-mediated cleavage of pcGAS is rapid and of high efficiency, which is reported in other literatures [23,32]. These findings indicate that SVV 3C WT interacts with pcGAS and the interaction is likely abolished upon 3C-mediated cleavage (Fig 6A).

Since SVV 3C cleaves pcGAS, we hypothesize that 3C viral protease likely co-localizes with its substrate pcGAS for cleavage in cells. To test this hypothesis, we performed microscopic co-localization analysis by the construction of a cell line stably expressing GFP-pcGAS in Hela cells via lentivirus over-expression system. We then transfected the empty vector, SVV 3C WT plasmids in Hela cells expressing GFP-pcGAS and detected the co-localization of SVV 3C and pcGAS at 12 h post transfection. The results showed that 3C WT protein (red) and pcGAS (green) were co-localized in the cytoplasm (Fig 6B). The dsDNA poly(dA:dT) is widely used as an agonist of cGAS for promoting the dimerization of cGAS [33]. Next, we co-transfected poly(dA:dT) with or without SVV 3C in Hela cells expressing GFP-pcGAS. We observed poly(dA:dT) induced significantly more co-localized spots of 3C and pcGAS (Fig 6C). To determine whether 3C also co-localizes with pcGAS upon SVV infection, we infected SVV (MOI = 10) in Hela cells or Hela cells expressing GFP-pcGAS. We observed more co-localization of 3C and pcGAS in the cytoplasm of Hela cells expressing GFP-pcGAS upon SVV infection (Fig 6D). Taken together, these data show that SVV 3C co-localizes with pcGAS in the cytoplasm of cells upon DNA transfection or SVV infection.

### The cleavage of pcGAS suppresses innate antiviral immunity

**Fig 7:**
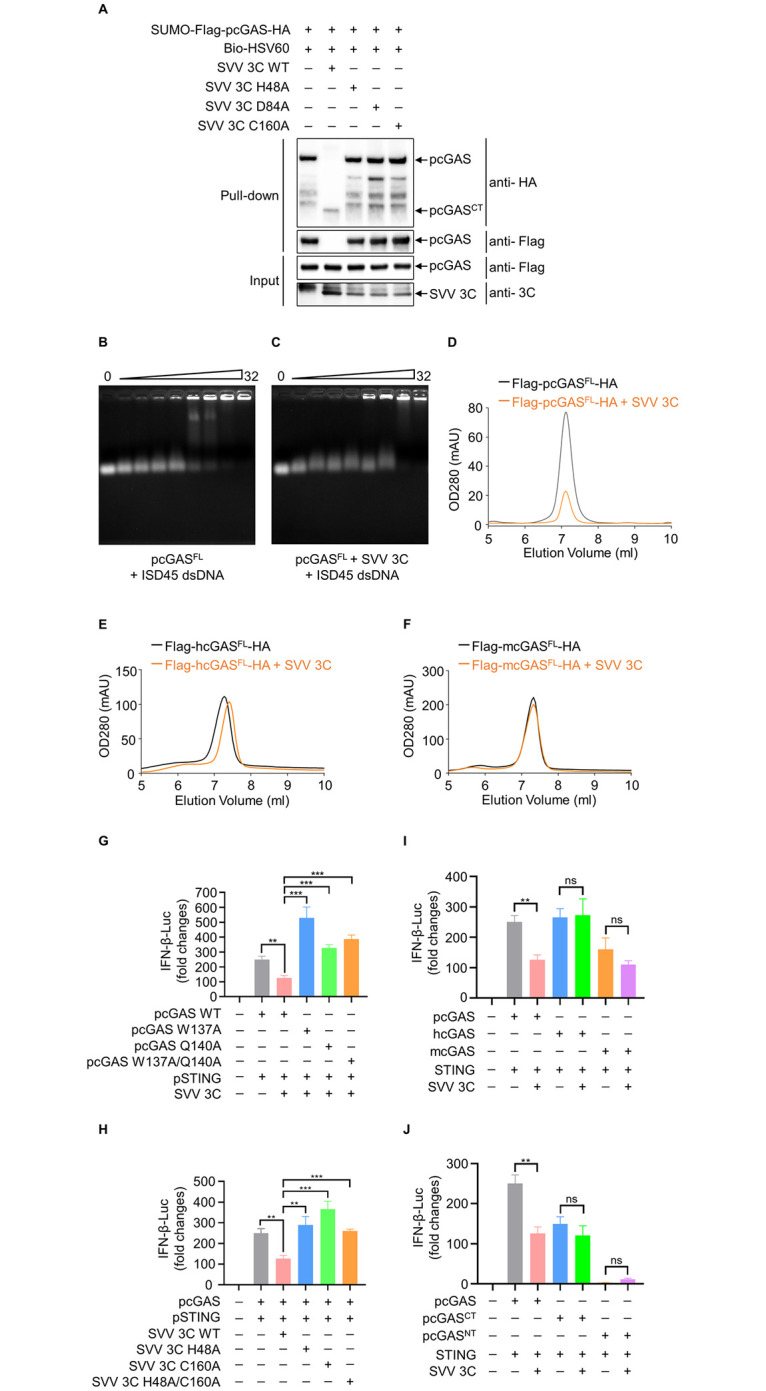
The cleavage of pcGAS suppresses innate antiviral immunity. **A**, Streptavidin pull-down assays of the binding of biotin-HSV-60 dsDNA to cell-free, recombinant pcGAS which was pre-incubated with wild-type 3C or 3C mutants. **B,** DNA binding analysis of full-length pcGAS in a reaction containing 45 bp interferon stimulatory DNA (ISD) and pcGAS with increased protein concentrations from 50 to 1600 μM at room temperature for 1 h followed by electrophoretic mobility shift assay (EMSA). **C,** DNA binding analysis of full-length pcGAS in a reaction containing 45 bp interferon stimulatory DNA (ISD), SVV 3C and pcGAS with increased protein concentrations from 50 to 1600 μM at molar ratios of 2:1 at room temperature for 1 h followed by EMSA. **D, E, F,** cGAS activity assay by ion exchange chromatography. 10 μM full-length pcGAS (**D**), hcGAS (**E**) or mcGAS (**F**) was incubated with the Salmon Sperm DNA in reaction buffer at 37°C for 2 h. The reaction product was first purified by ultrafiltration and then analyzed using a MonoQ ion exchange column. **G**, Detection the activation of IFN-β promoter in HEK-293T cells transfected with an IFN-β-Luc reporter plasmid, plus expression plasmid for pSTING and SVV 3C or expression plasmid for WT or mutant pcGAS for 24 h by dual-luciferase report assay. **H**, Detection the activation of IFN-β promoter in HEK-293T cells transfected with an IFN-β-Luc reporter plasmid, plus expression plasmid for pSTING and pcGAS or expression plasmid for WT or mutant SVV 3C for 24 h by dual-luciferase report assay. **I, J**, Detection the activation of IFN-β promoter in HEK-293T cells transfected with an IFN-β-Luc reporter plasmid, plus expression plasmid for pSTING and SVV 3C or expression plasmid for pcGAS, hcGAS or mcGAS (**I**) or expression plasmid for full-length pcGAS, N terminus or C terminus of pcGAS (**J**) for 24 h by dual-luciferase report assay. Results are presented relative to those of renilla luciferase (co-transfected as an internal control). Results are representative of three independent experiments. Means ± SD are shown in **E** (n = 3). Two-tailed unpaired *t*-test was used for the statistical analysis, **P < 0.01, ***P < 0.001. ns, no significance.

**Fig 8:**
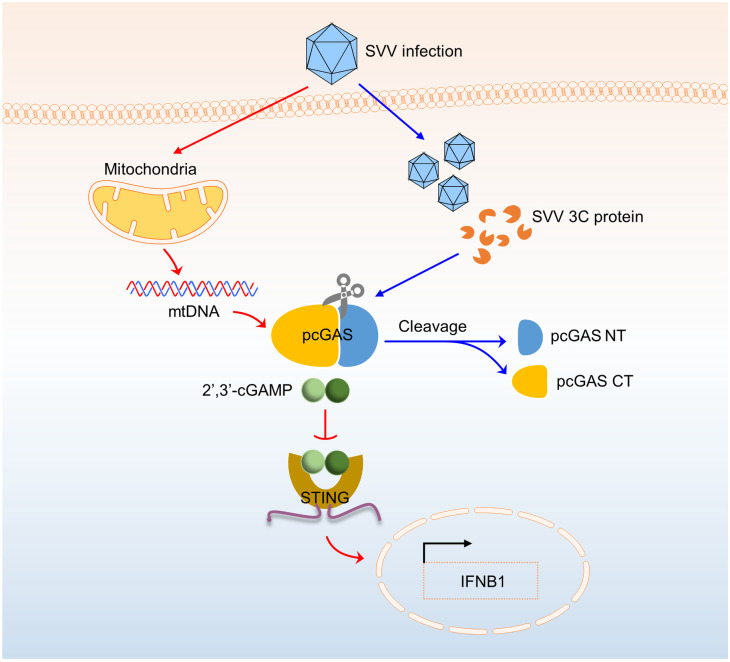
Schematic model of SVV protease 3C-mediated cleavage of pcGAS for immune evasion. SVV infection causes mtDNA release in the cytosol to activate cGAS-STING pathway. To evade the innate immune response, SVV evolves a strategy that disrupts pcGAS-mediated DNA binding, enzymatic activity and interferon signaling by specifically cleaving pcGAS within its N-terminal domain by viral protease 3C.

The N-terminal domain of cGAS is disordered and positively changed [34]. It is involved in DNA binding and promoting cGAS activation [35]. Interestingly, our data showed that SVV 3C cleaved within pcGAS NT. To determine the roles of pcGAS NT in DNA binding, we first conducted pulldown assay. We incubated pcGAS^FL^ with WT 3C protein or three mutant 3C proteins at 37°C for 2 h followed by DNA pulldown. The results suggested that co-incubation of WT 3C with pcGAS drastically reduced pcGAS binding to biotinylated HSV60 dsDNA in comparison with H48A, D84A and C160A mutant 3C (Fig 7A). To confirm this result, we did electrophoretic mobility shift assay (EMSA) assay for DNA binding to cGAS. We found pcGAS^FL^ bound to ISD45 on a dose-dependent manner (Fig 7B). However, when incubation of pcGAS^FL^ with WT 3C significantly reduced ISD45 binding (Fig 7C). Furthermore, we tested the effect of SVV 3C cleavage on the enzyme activity of pcGAS^FL^. First, we conducted enzymatic activity assays of pcGAS^FL^, hcGAS^FL^ and mcGAS^FL^ using a MonoQ ion exchange column (Fig 7D, 7E and 7F, black peak line). After adding 3C protein in the enzymatic reaction system, cGAMP synthesis significantly decreased by pcGAS^FL^, but not by hcGAS^FL^ and mcGAS^FL^, suggesting the cleavage of pcGAS^FL^ by SVV 3C reduced its catalytic activity (Fig 7D, 7E and 7F, yellow peak line). These data indicated that SVV 3C cleavage reduces the enzyme activity of pcGAS. To further confirmed the effect of pcGAS cleavage by SVV 3C on signaling transduction, we conducted a double-luciferase reporter assay to detect IFN-β promoter activation. The plasmids encoding pcGAS (WT, W137A, Q140A and W137A/Q140A) together with pGL3.0-IFN-β-Luc, pRL-TK, pcDNA3.1-pSTING and pcDNA3.1-SVV 3C were co-transfected in HEK-293T cells, respectively. Luciferase activity was detected at 24 h post transfection. The result showed that SVV 3C significantly inhibited the IFN-β reporter activation induced by WT pcGAS (Fig 7G). Similarly, we also tested the effect of WT 3C or 3C catalytic mutants on IFN-β promoter activation and the results showed that WT 3C rather than the catalytic mutants strongly inhibit IFN-β reporter activation (Fig 7H). Further experiments showed that SVV 3C specifically reduced pcGAS-mediated IFN-I reporter activation. However, SVV 3C has no notable effect on hcGAS and mcGAS-mediated IFN-I reporter activation (Fig 7I). Moreover, the activity of pcGAS^CT^ and pcGAS^NT^ were not affected by SVV 3C, indicating there is no other cleavage site in pcGAS^CT^ and pcGAS^NT^ (Fig 7J). To further confirm our findings using virus, we tried to generate SVV with 3C mutations by reverse genetic system. Construction of SVV infectious clone with 3C mutation was performed as shown in the schematic illustration (S9A Fig). However, we failed to generate either H48K or C160T SVV 3C mutation viruses, likely due to the essential roles of 3C in viral replication. We attempted to rescue viruses with more single residue substitutions in SVV 3C, which include mutations H75T, H78K, H79P, L85P, K198R and H202K. Finally, we successfully rescued two mutation SVV 3C viruses, named rH75T and rH78K. However, the H79P, L85P, K198R and H202K mutants failed to be rescued (S9B Fig). SVV mutant viruses rH75T and rH78K were able to infect BHK-21 cells, as shown by immunofluorescence (S9C Fig). Next, we tested whether 3C mutants H75T and H78K lost their ability to cleave pcGAS. Unfortunately, these two 3C mutants still cleave pcGAS just as WT SVV 3C (S9D Fig). Collectively, these studies demonstrate that SVV 3C specifically cleaves pcGAS but not hcGAS and mcGAS to suppress pcGAS-mediated DNA binding, enzymatic activity, and interferon induction (Fig 8).

## Discussion

The detection of viral DNA is a central strategy by which the host sense infection and launches protective immune responses. The enzyme cGAS is activated by dsDNA and catalyzes the synthesis of a noncanonical cyclic dinucleotide cGAMP that binds and activates the receptor STING to induce the expression of type-I interferons. Besides its primary roles in fighting against DNA virus infection, cGAS is a potent restriction factor for RNA viruses, indicating a crosstalk between innate sensing of cytosolic DNA and RNA [13,36]. As early as 2011, Charles Rice’s group used an expression screening approach to identify C6orf150 (cGAS) as one of the most targeted antiviral molecules for restricting positive-sense single-stranded RNA viruses (+ssRNA), including flaviviruses, alphaviruses. Importantly, mortality and virus replication were increased in cGAS-knock-out mice infected with West Nile virus (WNV) [37].

While cGAS could bind to cytoplasmic RNA and promote liquid droplets *in vitro*, RNA could not induce the correct conformational change of cGAS to synthesize cGAMP [38]. Since cGAMP is required for STING activation, it remains mysterious how cGAS contributes to RNA virus sensing. Pattern recognition receptors (PRRs) could not only recognize pathogen molecular patten (PAMP) but also detect danger associated molecular patten (DAMP), such as mtDNA [39]. Cellular stress can induce aberrant mtDNA packaging to promote cGAS-STING dependent signaling and induce interferon expression [40]. Two previous studies showed that dengue virus infection caused mtDNA release into the cytosol [15,16]. It was also shown that IAV viroporin M2 and EMCV viroporin 2B were reported to induce mitochondrial dysfunction and mtDNA release [17]. Moreover, CHIKV infection caused distinct puncta of extranuclear DNA [18]. In addition to human viruses, PRRSV infection could also cause mtDNA release into cytosol and co-localized with cGAS, suggesting a broad spectrum of mtDNA leakage can be caused by RNA virus infection [19].

Due to virus-host coevolution in nature, RNA viruses employ a variety of strategies to overcome cGAS-STING mediated immune response [13]. For example, DENV NS2B protease targets cGAS for lysosomal degradation to dampen cGAS activation [15]. Recently, DENV NS2B3 also was reported to cleave hcGAS NT, suggesting different ways of DENV targeting cGAS for immune evasion [41]. Another study showed that DENV1-4 cleaved human STING within and RG motif between residues 78 and 79 but does not target non-human primates STING [25]. Furthermore, ZIKA non-structural protein NS1 recruits USP8 to stabilize caspase-1 leading to cGAS cleavage, albeit via an indirect strategy [20]. Zika virus NS2B3 protease cleaves hSTING but not mSTING in a species-specific fashion [24]. Besides flaviviruses, CHIKV, an alphavirus, utilizes the capsid protein to target cGAS for degradation via an ATG7-dependent autophagic mechanism to inhibit damage DNA induced type-I interferon expression in primary human cells [18].

It was reported that EMCV infection stimulated mtDNA release in HEK-293FT cells via viroporin 2B. A recent study showed that picornaviruses, including EV-A71, SVV and FMDV, activate cGAS by triggering mtDNA release *in vitro and vivo*. SVV 2C was responsible for suppressing cGAS expression via the autophagy pathway [21]. In addition, they also showed that EV-A71, SVV and FMDV 2C interacted with STING and inhibited STING activation. Interestingly, they infected PK-15 cells with SVV and found a dose-dependent decreasing of cGAS expression but no effect on STING expression was observed, which is consistent with our data. Similarly, this study also showed that EV-A71 infection did not affect cGAS expression in Hela and HT-29 cells the same results as we tested in Hela and RD cells.

In conclusion, this study showed that RNA virus protease directly cleaved cGAS to suppress innate immune signaling in species-specific manner. It was surprising that SVV 3C, but not the other picornaviruses 3C, cleaves cGAS. The reason for this is that SVV 3C has very low sequence homology and a far evolutionary distance from other picornaviruses 3C. Another open question is why SVV 3C and DENV NS2B3 specifically cleave cGAS NT but not the catalytic domain. It was reported that hcGAS and mcGAS NT enhance DNA binding, enzymatic activity, and oligomerization of cGAS [34]. On the other hand, cGAS NT contains a lot of positive charged residues, which could enhance cGAS-DNA phase separation by increasing DNA binding. Increasing evidence shows that RNA viruses are capable to target the cGAS-STING pathway to evade innate immune surveillance. Our findings provide important insights for developing antiviral strategies against picornaviruses and may have critical clinical implications for controlling cGAS-related diseases.

## Materials and methods

### Cells and virus

HEK-293T cells (Human embryonic kidney cells), ST cells (Swine testis cells), PK-15 cells (Porcine kidney-15), Hela cells, RD cells (Rhabdomyosarcoma cells) and BHK-21 cells (Baby hamster kidney-21) were cultured in Dulbecco’s Modified Eagle Medium (DMEM) (MACGENE, #CM10013) containing 10% fetal bovine serum (FBS) (PlantChemMed, #PC-00001) at 37°C with 5% CO_2_. HEK-293T cell lines stably expressing pcGAS-pSTING or pcGAS were constructed using PiggyBac Transposon system and detected by the microscope (Falcon S300, Alicelligent). Hela cell line stably expressing pcGAS was constructed using lentivirus over-expression system. The SVV was stored in our laboratory. EV-A71 was provided by Dr. Peng Sun from Wenzhou Medical University.

### Antibodies

The following antibodies were used in this study. Rabbit anti-HA monoclonal antibody (MAb) (#3724), rabbit anti-Myc MAb (#2278), anti-mouse IgG HRP-linked antibody and anti-rabbit IgG HRP-linked antibody were purchased from Cell Signaling Technology (Beverly, MA). Mouse anti-FLAG MAb (#F1804) was obtained from Sigma-Aldrich (MO, USA). Rabbit anti-GAPDH polyclonal antibody (PAb) (#10494-1-AP) and rabbit anti-cGAS PAb (#26416-1-AP) were purchased from Proteintech Group (Chicago, IL). ALexa Flour 633 goat anti-mouse IgG(H+L) (#A21052) was obtained from Thermo Fisher Scientific (Waltham, MA, USA). Mouse anti-SVV 3C polyclonal Ab was prepared by our laboratory.

### Plasmids

The N-terminal Myc/C-terminal Hemagglutinin (HA) double-tagged cGAS was ligated to pcDNA3.1 vector at the sites of *HindIII* and *EcoRI* in frame. The N-terminal FLAG/C-terminal HA double-tagged cGAS was ligated to His_6_-SUMO-pET28a vector at the sites of *BamHI* and *HindIII*. N-terminal FLAG tagged 3C proteases of picornaviruses were ligated to pcDNA3.1 or 3×FLAG-CMV-10 vector. The 3C protease of SVV was ligated to His_6_-SUMO-pET28a vector. Mutant plasmids of cGAS and SVV 3C protease were constructed according to the manufacturer’s instructions of Mut Express II Fast Mutagenesis Kit V2 (Vazyme, #C1002).

### Detection of SVV replication

For activation of cGAS-STING signaling pathway, ST cells were stimulated with different dose poly(dA:dT) (InvivoGen, #tlrl-patn, tlrl-patn-1) or 2’,3’-cGAMP (InvivoGen, #tlrl-nacga23) for 6 h prior to virus infection. ST cells were then infected with SVV (MOI = 0.1) for different time points to determine the gene copies of SVV. RNA was extracted from whole-cell lysates with RNAsimple Total RNA Kit (TIANGEN, #DP419) and reversely transcribed to cDNA with HiScript II Q RT SuperMix for qPCR (+gDNA wiper) Kit (Vazyme, #R223-01). The qPCR was performed using ChamQ SYBR qPCR Master Mix (Vazyme, #Q712-02). Threshold cycle numbers were normalized to triplicate samples amplified with primers specific for GAPDH.

### RNA interference

PK-15 cells were transfected with siRNAs by RNAiMAX transfection reagent (Invitrogen, # 13778150) for 36 h before SVV infection. The knockdown efficiency was detected through RT-qPCR.

### Generation of cGAS knockout cell line via CRISPR/Cas9

The CRISPR sgRNAs targeting *pcGAS* were designed [42] and ligated into the Lenti-CRISPRv2 plasmid. The Lenti-CRISPRv2 plasmid (with sgRNA cloned) was co-transfected into HEK-293T cells with the packaging plasmids psPAX2 and pMD2.G for 48~72 h to generate lentivirus. ST cells were infected with the recombinant lentivirus for 24 h, followed by selection with puromycin (5 μg/mL) for 3~5 days. The positive cells were subcloned into 96-well plates for single-clone growth and then determined by sequencing.

### Purification and quantification of cytoplasmic mtDNA

HEK-293T cells or PK-15 cells were plated in 10 cm dish and infected with SVV (MOI = 10) when cell confluence was up to 100%. Cells were harvested at different time points post infection. A quarter of the cells from each sample were used for RNA extraction with RNAsimple total RNA kit, and the other cells were used for isolating cytoplasmic supernatant without mitochondria using mitochondria isolation kit (Thermo Fisher scientific, #89874). QIAamp DNA Mini and Blood Mini kit (QIAGEN, #51306) was used for purifying and collecting DNA from supernatant. Meanwhile, total RNA corresponding to each sample was obtained by RNAsimple total RNA kit and reversely transcribed into cDNA. Specific DNA fragments were quantified by qRT-PCR, and GAPDH corresponding to cDNA of each sample served as the internal control for calculating relative values of mtDNA. The sequences of primers were as follows: hGAPDH-F: CTCTGCTCCTCCTGTTCGAC, hGAPDH-R: AATCCGTTGACTCCGACCTT; hmtDNA-F: CTATCACCCTATTAACCACTCA, hmtDNA-R: TTCGCCTGTAATATTGAACGTA; pGAPDH-F: ACATGGCCTCCAAGGAGTAAGA, pGAPDH-R: GATCGAGTTGGGGCTGTGACT; pmtDNA-F: ACAGCTGCACTACAAGCAATGC, pmtDNA-R: GGATGTAGTCCGAATTGAGCTGATTAT.

### Protein expression and purification

The sequences encoding full-length, catalytic domain and mutant catalytic domain (W137A/Q140A) of pcGAS, full-length hcGAS and mcGAS, SVV 3C WT and its mutants (H48A, D84A and C160A), EV-A71 3C were cloned into His_6_-SUMO-pET28a vector followed by transformation into E.coli BL21(DE3) (TransGen Biotech, #CD601). The bacteria were cultured in LB medium with appropriate Kanamycin. When OD600 reached at 1.0, the proteins were induced overnight at 16°C with 1 mM isopropyl β-D-1-thiogalactopyranoside (IPTG, #18070). The bacteria were lysed in buffer containing 50mM Tris-HCl (pH 8.0), 300 mM NaCl. The proteins were then purified by Ni-NTA beads followed by washing with the buffer containing 20 mM Tris-HCl (pH 7.5), 500 mM NaCl and 25mM imidazole and eluting with the buffer containing 20 mM Tris-HCl (pH 7.5), 150 mM NaCl and 250mM imidazole. The His_6_-SUMO tag was removed by SUMO protease overnight at 4°C. After that, the proteins were further purified by Hiload 16/600 Superdex 200pg gel-filtration chromatography with running buffer containing 20 mM Tris-HCl (pH 7.5), 150 mM NaCl and then purified by resource S ion exchange with buffer A containing 10 mM Tris-HCl (pH 8.0), 100 mM NaCl and buffer B containing 10 mM Tris-HCl (pH 8.0), 1 M NaCl. The purified proteins were concentrated to ~12 mg/ml and frozen in liquid nitrogen immediately. All purified proteins were stored in running buffer with 5 mM DTT.

### Detection of pcGAS cleavage in cells

HEK-293T cells were seeded into 6-well plates. When the cells reached 80% confluent, a plasmid encoding Myc-pcGAS-HA (2 μg), Myc-hcGAS-HA (2 μg) or Myc-mcGAS-HA (2 μg) and a plasmid encoding FLAG-SVV 3C (1.5 μg) were co-transfected for 24 h with Lipo2000 (Invitrogen, #11668019). Cells were then lysed with NP-40 (Solarbio, #N8032) containing protease inhibitors (Solarbio, # P6730) and analyzed by Western blot. The proteins in the polyacrylamide gel were transferred to polyvinylidene fluoride (PVDF) membranes (Millipore, #ISEQ00010) and blocked with PBS containing 5% skim milk for 2 h. And blots were incubated with anti-FLAG, anti-Myc and anti-HA to detect the expression levels of SVV 3C, cGAS N terminus and cGAS C terminus, respectively, followed by incubation with anti-mouse IgG HRP-linked secondary antibody or anti-rabbit IgG HRP-linked secondary antibody. The results were analyzed by GelView 6000Plus (Guangzhou Biolight Biotechnology). The co-transfection of plasmids encoding hcGAS and EVA-71 3C were done according to the above method. HEK-293T cells stably expressing pcGAS alone or both pcGAS and pSTING were plated in 12-well plates and infected with SVV when cells grown to 100% confluence. The cell culture medium was discarded, and the cells were washed three times with PBS. Next, cells were incubated with SVV (MOI = 0.1 or 10) at 37°C for 1 h. The surplus virus was washed off with PBS and the cells were cultured with 2% FBS DMEM. Finally, cells were harvested at different time points after infection and lysed by NP-40 buffer with protease inhibitor followed by Western blot.

### In vitro cleavage assay and mass spectrometry

For the cleavage assay *in vitro*, 20 μg SUMO-pcGAS^FL^, SUMO-hcGAS^FL^, or SUMO-mcGAS^FL^ recombinant protein incubated with different amounts of purified SVV 3C protein (0.01, 0.05, 0.1, 0.5, 1, 5, 10 μg) in a 25-μl reaction containing 50 mM HEPES (PH 7.5), 3 mM EDTA, 150 mM NaCl, 0.005% (vol/vol) Tween-20 and 10 mM DTT at 37°C for 2 h. The cleavage reaction was terminated by adding SDS-PAGE loading dye. The reaction mixtures were analyzed by SDS-PAGE and stained with coomassie blue dye for 10 min. The major cleavage product band with ~40 kDa was excised form the SDS-PAGE gel and digested by trypsin protease for mass spectrometry. The mass spectrometry results were analyzed by Matrix Science.

### Circular dichroism (CD) assay

The purified SVV 3C protein and its mutants were diluted 100-fold with 10 mM Tris-HCl to obtain 0.2 mg/ml protein and loaded into a quartz cuvette with a path length of 0.01 cm for measurement. First, the buffer containing only 10mM Tris-HCl was used for subtraction of the baseline signal. To investigate the exact composition of SVV 3C and its mutants, circular dichroism spectra (190 nm to 260 nm) of the proteins were measured by Chirascan plus (Applied Photophysics Ltd) at 25°C. For measuring the stability of SVV 3C WT and its mutants, samples of the same concentration were tested at different temperatures (25°C, 35°C, 45°C and 55°C). Each sample was repeated three times. Then, the data were deconvoluted via CDNN software and analyzed by GraphPad Prism.

### Co-immunoprecipitation (Co-IP)

HEK-293T cells grown in 6 cm dish were transfected with the indicated plasmids by using Lipo2000. At 24 h post-transfection, cells were collected and lysed with NP-40. After centrifugation for 10 min at 12,000 rpm, supernatants were immunoprecipitated with mouse anti-FLAG MAb and protein A/G plus-agarose (Santa Cruz Biotechnology, #sc-2003). After 6 h of incubation, beads were washed three times with ice-clod PBS. Immunoprecipitates or whole cell lysates were boiled with SDS sample buffer, separated by SDS-PAGE, transferred onto PVDF membranes, and then blotted with specific antibodies.

### Confocal microscopy

Hela cells stably expressing GFP-cGAS (GFP-cGAS-Hela) were seeded into 24-well plates containing slides. When confluence reached up to 40–50%, the cells were transfected with FLAG-SVV 3C plasmid upon poly(dA:dT) stimulation or infected with SVV (MOI = 10). After 12 h, the supernatant was discarded and the cells were washed with PBS. After treatment with 4% fixative solution (Solarbio, #P1110) for 30 min and 0.5% Triton X-100 for 10 min, the cells were washed with PBS and blocked with 1% bovine serum albumin (BSA) (Sigma-Aldrich, #A7906-100G) for 30 min. After completion, the cells were incubated with anti-FLAG antibody for 2 h and washed three times with PBS, followed by incubation with ALexa Flour 633 goat anti-mouse IgG (H+L) antibody for 30 min and treated with 0.1 μg/ml 4’,6-diamidino-2-phenylindole (DAPI) (Beyotime, #C1002) for 5 min. After washing with PBS, the stained cells were observed using a Nikon A1 confocal microscope. Images were collected and analyzed by NIS-Elements AR.

### Electrophoretic mobility shift assays (EMSA)

For the DNA binding assay, 50 μM of the 45 bp interferon stimulatory DNA (ISD45), 5’-TACAGATCTACTAGTGATCTATGACTGATCTGTACATGATCTACA-3’ (Synthesized by Sangon Biotech), was mixed with full-length pcGAS at molar ratios of 1:1, 1:2, 1:4, 1:8, 1:12, 1:16, 1:24, 1:32. The mixtures were resolved on 1% agarose gel using an electrophoresis buffer of 40 mM Tris-HCl (pH 9.2) at constant voltage of 110 V. To determine the effect of SVV 3C on dsDNA binding to pcGAS, the mixture including SVV 3C and full-length pcGAS with the molar ratio of 2:1 was incubated at room temperature for 1 h. ISD45 was added to the mixture and then were resolved using 1% agarose gel. The gels were stained with ethidium bromide and documented using GelView 6000Plus.

### The cGAS activity assay

The 10 μM full-length pcGAS, hcGAS and mcGAS were incubated with the Salmon Sperm DNA (Thermo Fisher scientific, #15632011) in reaction buffer containing 20 mM HEPES (pH 7.5), 5 mM MgCl_2_, 2 mM ATP and 2 mM GTP at 37°C for 2 h. Samples were centrifuged at 12,000 rpm for 10 min. The products in the supernatant were separated by ultrafiltration. The products were further analyzed by MonoQ ion exchange column (GE Healthcare) with running buffer containing 50 mM Tris-HCl (pH 8.5) and followed by elution by gradient NaCl running buffer. The cGAS products (2’,3’-cGAMP) were analyzed by ion exchange chromatography.

### DNA pulldown assay

The 10 μg recombinant pcGAS protein was incubated with 5 μg WT 3C or 3C mutant proteins in the buffer containing 50mM HEPES (PH 7.5), 3mM EDTA, 150mM NaCl, 0.005% (vol/vol) Tween-20 and 10mM DTT at 37°C for 2 h. 3 μg biotinylated HSV60 dsDNA (Synthesized by Sangon Biotech) was pre-incubated with 20 μl streptavidin magnetic beads (Changzhou Smart-Lifesciences Biotechnology, #SM017005) for 2 h. The mixture containing pcGAS and 3C protein were then added to the biotinylated HSV60 bound beads and incubated for 4 h. After washing and denaturation, the proteins bound to beads were subjected to western blot.

### Reverse genetic manipulation

The SVV infectious clone was used as the amplification template, the mutation was introduced on 3C by primers, and the in-fusion clone method was used for cloning. The PCR primers for mutation sites include forward primers for insertion fragments and reverse primers for insertion fragments. The 5 ’ end of the primers contains an 18 nt sequence homologous to the end of the adjacent fragment (insertion fragment or vector). Age I and Mlu I were used to digest the SVV infectious clone. The end primers of the vector included forward primer and reverse primer. The 5 ’ end of the primer had 18 nt homologous sequences with the end of the vector. Fragment 1 was amplified using the vector forward primer and the insertion fragment reverse primer, and fragment 2 was amplified using the insertion fragment forward primer and the vector reverse primer. The digested vector and amplified DNA fragments were purified by gel, and Hi-Fusion Cloning Mix V2 (Monad, #MC40101M) recombined the purified fragments and vectors at 50°C for 45 min. After the reaction, the mutant plasmids were transformed into E.coli DH5α cells and grown overnight at 37°C in the presence of ampicillin. The rescued mutant viruses were subjected to RT-PCR for sequencing to confirm the presence of amino acid substitution and the absence of other changes.

### IFN-β reporter assay

HEK-293T cells were seeded into 24-well plates. When the cells reached 80% confluent, pGL3.0-IFN-β-Luc (300 ng), pRL-TK (30 ng), porcine cGAS (200 ng), porcine STING (100 ng), and SVV 3C protease (200 to 600 ng) plasmids were co-transfected using Lipo8000 (Beyotime Biotechnology, #C0533-0.5ml). Samples were collected at 20 or 24 h post-transfection and lysed with 1×passive lysis buffer. After centrifugation, 20 μl supernatant was added to 96-well plate, and the activities of firefly and renilla luciferase were measured according to the Dual-Luciferase Reporter Assay System (Promega, #E1960).

### Statistical data analysis

All the graphs and relevant statistical tests used in the work were created by GraphPad Prism version 8.0.1. Data were expressed as mean ± SD and statistically analyzed with a two-tailed unpaired Student’s *t*-test. A *P* value of < 0.05 was considered to be statistically significant. *P < 0.05; **P < 0.01 ***P < 0.001. ns, no significance.

## Supporting information

S1 Fig Poly(dA:dT) and 2’,3’-cGAMP transfection reduces SVV transcription. **A-D,** The RT-qPCR analysis of SVV 3C (**A, C**) and VP1 (**B, D**) mRNA expression (related to GAPDH) in ST cells mock-transfected or transfected with poly(dA:dT) or 2’,3’-cGAMP for 6 h in a dose-dependent manner, followed by infection with SVV (MOI = 0.1) for another 0, 6, 12 and 24 h. Results are representative of three biological replicates. Means ± SD are shown in **A-D** (n = 3). Two-tailed unpaired *t*-test was used for the statistical analysis, ***P < 0.001.
(TIF)

S2 Fig SVV infection induces the release of mtDNA into the cytosol. **A, B**, HEK-293T (**A**) or PK-15 (**B**) cells were infected with SVV (MOI = 10), and the cells were harvested at 0, 6, 9, 12, 15 and 18 h post-infection. The cytoplasmic lysates without mitochondria were isolated using mitochondrial extraction kit, followed by extraction of mtDNA in cytoplasm using QIAamp DNA Mini and Blood Mini kit. The relative mtDNA abundances were analyzed through detecting expression of mtDNA relative to GAPDH by qPCR. Results are representative of three biological replicates. Means ± SD are shown in **A, B** (n = 3). Two-tailed unpaired *t*-test was used for the statistical analysis, **P < 0.01, ***P < 0.001.
(TIF)

S3 Fig The cGAS and picornavirus protease 3C recombinant proteins are purified. **A-D,** Recombinant proteins SUMO-Flag-pcGAS^FL^-HA (**A**), SUMO-Flag- hcGAS^FL^-HA (**B**), SUMO-Flag-mcGAS^FL^-HA (**C**) and SUMO-pcGAS^CAT^ (**D**) were purified by fast protein liquid chromatography (FPLC) and ion exchange, followed by SDS-PAGE analysis. **E, F,** The SUMO tag of recombinant SUMO-SVV wild-type 3C and SUMO-EV-A71 3C was removed by SUMO protease overnight at 4°C. The recombinant proteins SVV wild-type 3C and EV-A71 3C were purified by FPLC and ion exchange (**E**) or FPLC (**F**).
(TIF)

S4 Fig EV-A71 protease 3C does not cleave pcGAS, hcGAS and mcGAS. **A-C**, Western blot analysis of pcGAS, hcGAS or mcGAS cleavage and EV-A71 3C expression in HEK-293T cells transfected with 1.5 μg FLAG-EV-A71 3C plasmid and 2 μg wild-type Myc-pcGAS-HA (**A**), Myc-hcGAS-HA (**B**), or Myc-mcGAS-HA (**C**) plasmid for 24 h using anti-Myc, anti-HA and anti-Flag antibodies, respectively. **D-F,** SDS-PAGE analysis of *in vitro* cleavage of pcGAS, hcGAS or mcGAS in a 25-μl reaction containing 10 μg SUMO-pcGAS^CAT^ (**D**), SUMO-hcGAS^FL^ (**E**) or SUMO-mcGAS^CAT^ (**F**) recombinant protein with different amounts of purified recombinant protein EV-A71 3C (0.01, 0.05, 0.1, 0.5, 1, 5, 10 μg) for 2 h at 37°C.
(TIF)

S5 Fig The hcGAS is resistant to cleavage by HAV, CVB3, PV and HRV protease 3C. **A-D,** Western blot analysis of hcGAS cleavage and 3C expression in HEK-293T cells transfected with 2 μg wild-type Myc-hcGAS-HA plasmid and 1.5 μg FLAG-tagged plasmid encoding HAV 3C (**A**), 3C from CVB3-28 virus (**B**), 3C from Human poliovirus 3 strain Sabin 3 (**C**) or 3C from HRV-A16 virus (**D**) for 24 h using anti-Myc, anti-HA and anti-Flag antibodies.
(TIF)

S6 Fig The hcGAS resists cleavage by SVV and EV-A71 infection on endogenous level. **A**, Hela cells were infected with SVV (MOI = 10) and harvested at 0, 6, 12, 24, 30 and 36 h post-infection. Cytopathic effect (CPE) in Hela cells without or with SVV infection for 30 h was shown by microscopy. The expression of endogenous hcGAS and SVV 3C was analyzed by Western blot. **B**, Hela cells were infected with EV-A71 (MOI = 10) and collected at 0, 6 and 12 h post-infection. Cytopathic effect (CPE) in Hela cells without or with EV-A71 infection for 12 h was shown by microscopy. The expression of endogenous hcGAS was detected by Western blot using anti-cGAS antibody. **C,** RD cells were infected with EV-A71 (MOI = 10) and collected at 0, 6 and 12 h post-infection. Cytopathic effect (CPE) in RD cells without or with EV-A71 infection for 12 h was shown by microscopy. The expression of endogenous hcGAS was analyzed by Western blot using anti-cGAS antibody.
(TIF)

S7 Fig The mutant pcGAS and SVV 3C recombinant proteins are purified. **A,** The SUMO-pcGAS^CAT^ mutant (W137A/Q140A) recombinant protein was purified by FPLC and ion exchange, followed by SDS-PAGE analysis. **B-D,** The SUMO-SVV 3C H48A (**B**), SUMO-SVV 3C D84A (**C**) and SUMO-SVV 3C C160A (**D**) mutant recombinant proteins were cleaved by SUMO protease overnight at 4°C. Then these mutant recombinant proteins were purified by FPLC and ion exchange, followed by SDS-PAGE analysis.
(TIF)

S8 Fig The stability of SVV 3C WT and its mutants. **A-D,** The purified SVV 3C protein and its mutants were diluted 100-fold with 10 mM Tris-HCl to obtain 0.2 mg/ml protein. Circular dichroism spectra (190 nm to 260 nm) of SVV 3C WT (**A**), 3C H48A (**B**), 3C D84A (**C**) and 3C C160A (**D**) proteins were tested at different temperatures (25°C, 35°C, 45°C and 55°C).
(TIF)

S9 Fig SVV 3C with catalytic mutations fail to be rescued by reverse genetic manipulation. **A,** schematic diagram showing construction of plasmids with mutated 3C Protease. **B, C,** Rescue of 3C mutant virus. We tried to mutate the 3C residues listed in the table (**B**), and transfected SVV infectious clone with 3C mutant into cells (**C**). Tick means successfully rescuing the 3C mutant virus (**B**). Successfully rescued virus carried GFP fluorescence (**C**). **D,** Western blot analysis of pcGAS cleavage and SVV 3C expression in HEK-293T cells transfected with 2 μg wild-type pcGAS and 1.5 μg wild-type or mutant SVV 3C (3C WT, 3C H75T or 3C H78K) expression plasmid for 24 h using anti-Myc, anti-HA and anti-Flag antibodies.
(TIF)
