# Supplementary figures and images for: Species-specific cleavage of cGAS by picornavirus protease 3C disrupts mitochondria DNA-mediated immune sensing
Source: PLoS Pathog. 2023 Sep 14;19(9):e1011641. doi: 10.1371/journal.ppat.1011641 (PMC10521975; doi:10.1371/journal.ppat.1011641)

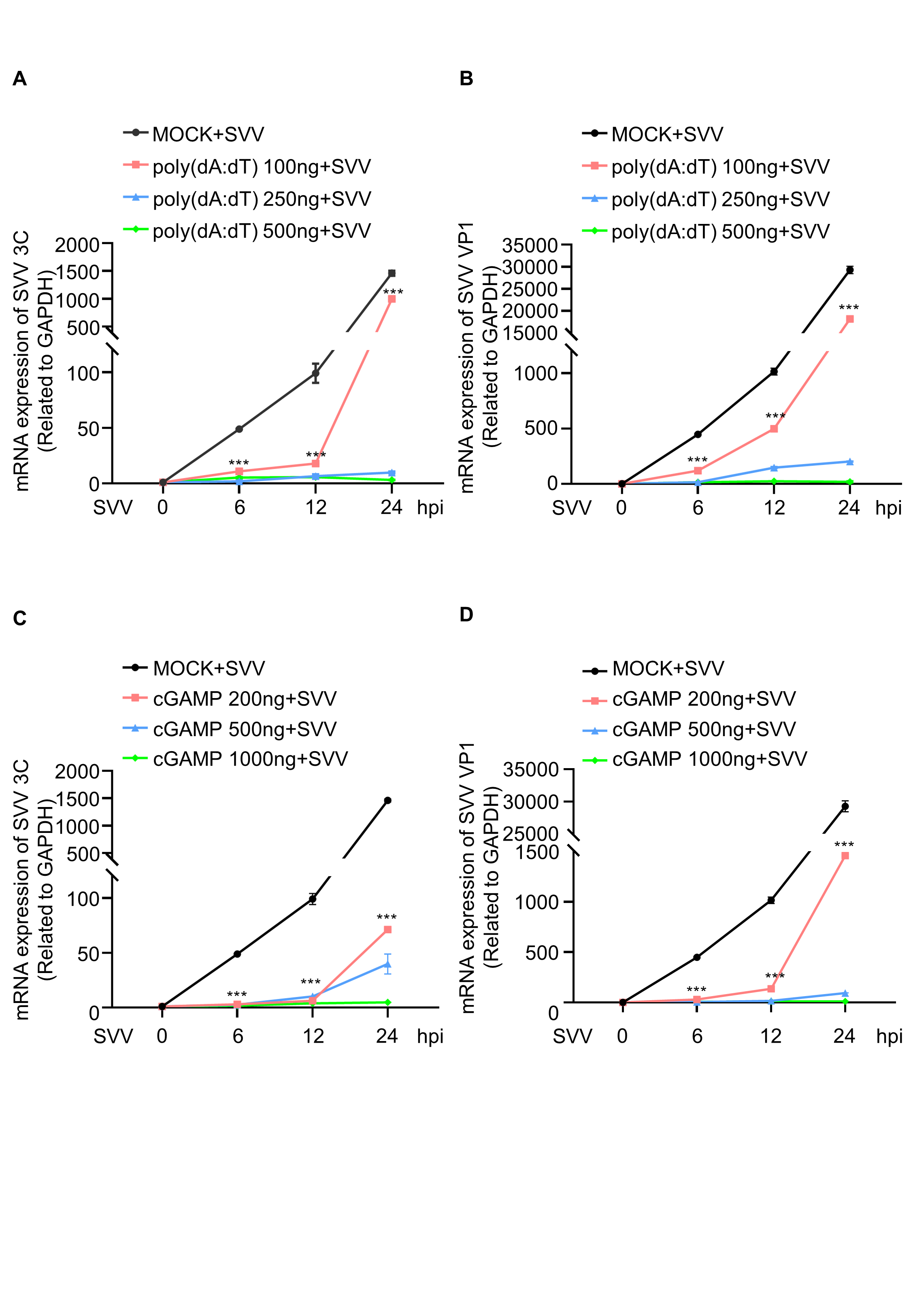

Supplement: S1 Fig — A-D, The RT-qPCR analysis of SVV 3C (A, C) and VP1 (B, D) mRNA expression (related to GAPDH) in ST cells mock-transfected or transfected with poly(dA:dT) or 2’,3’-cGAMP for 6 h in a dose-dependent manner, followed by infection with SVV (MOI = 0.1) for another 0, 6, 12 and 24 h. Results are representative of three biological replicates. Means ± SD are shown in A-D (n = 3). Two-tailed unpaired t-test was used for the statistical analysis, ***P < 0.001. (TIF) [file ppat.1011641.s001.tif]

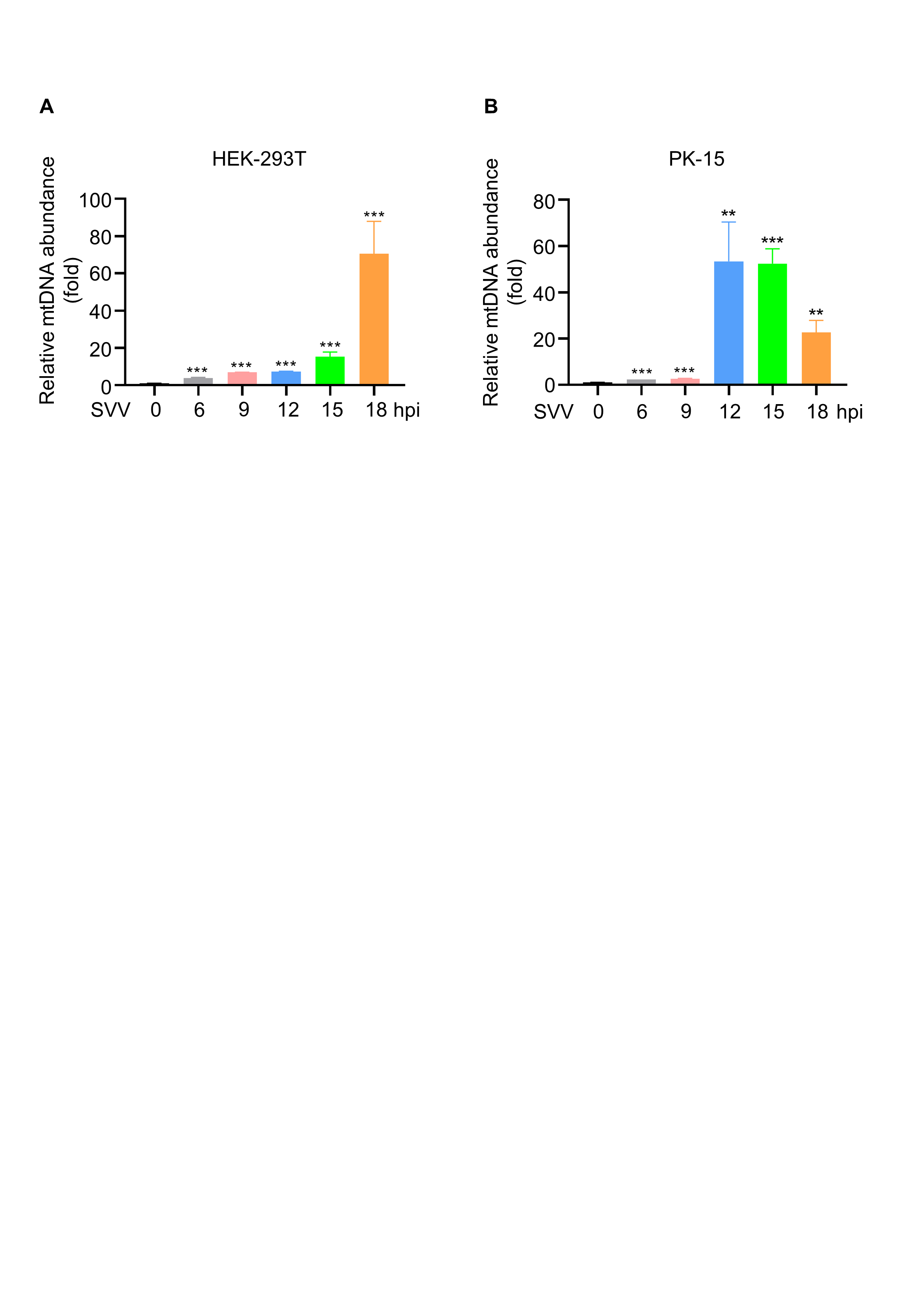

Supplement: S2 Fig — A, B, HEK-293T (A) or PK-15 (B) cells were infected with SVV (MOI = 10), and the cells were harvested at 0, 6, 9, 12, 15 and 18 h post-infection. The cytoplasmic lysates without mitochondria were isolated using mitochondrial extraction kit, followed by extraction of mtDNA in cytoplasm using QIAamp DNA Mini and Blood Mini kit. The relative mtDNA abundances were analyzed through detecting expression of mtDNA relative to GAPDH by qPCR. Results are representative of three biological replicates. Means ± SD are shown in A, B (n = 3). Two-tailed unpaired t-test was used for the statistical analysis, **P < 0.01, ***P < 0.001. (TIF) [file ppat.1011641.s002.tif]

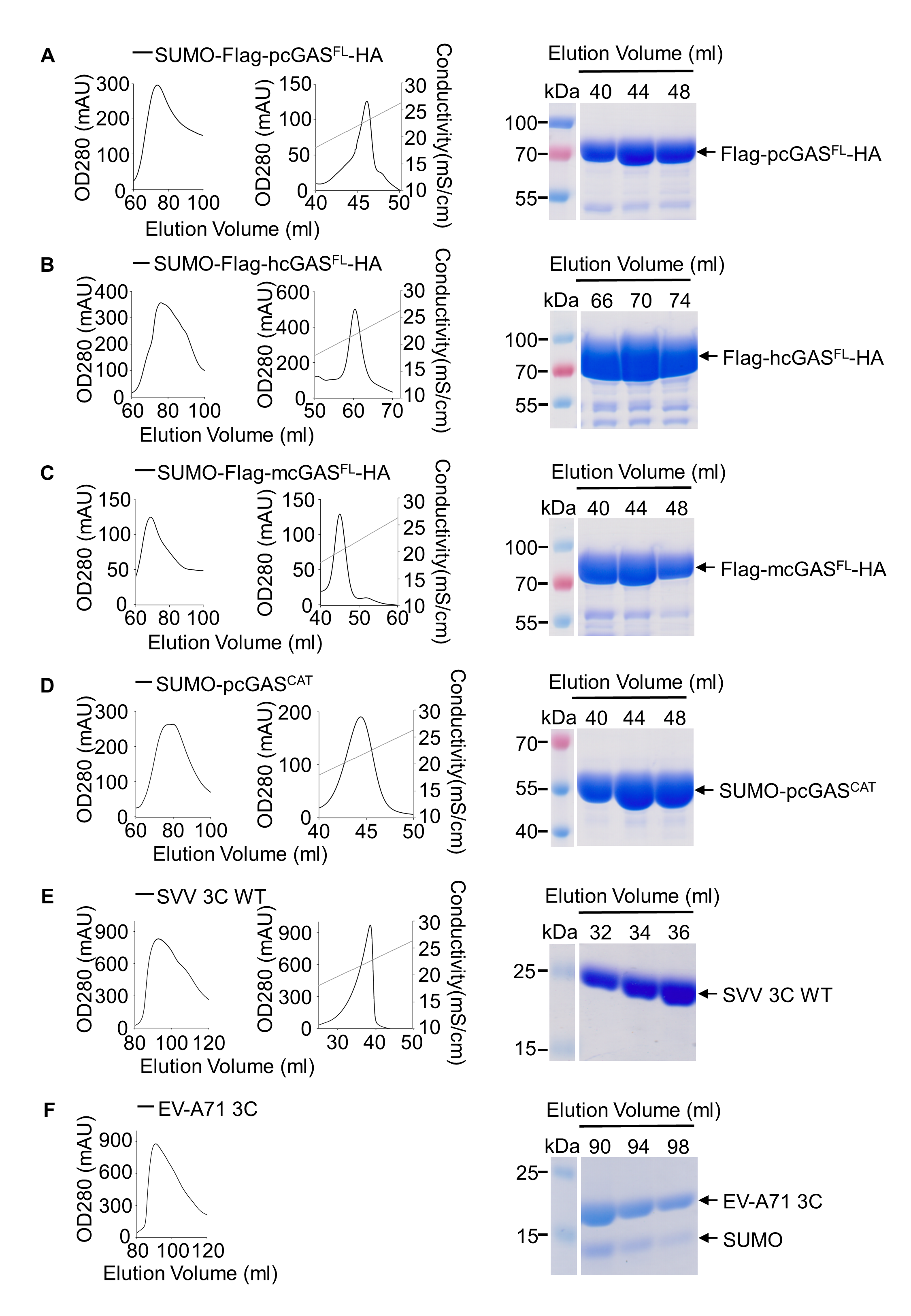

Supplement: S3 Fig — A-D, Recombinant proteins SUMO-Flag-pcGASFL-HA (A), SUMO-Flag- hcGASFL-HA (B), SUMO-Flag-mcGASFL-HA (C) and SUMO-pcGASCAT (D) were purified by fast protein liquid chromatography (FPLC) and ion exchange, followed by SDS-PAGE analysis. E, F, The SUMO tag of recombinant SUMO-SVV wild-type 3C and SUMO-EV-A71 3C was removed by SUMO protease overnight at 4°C. The recombinant proteins SVV wild-type 3C and EV-A71 3C were purified by FPLC and ion exchange (E) or FPLC (F). (TIF) [file ppat.1011641.s003.tif]

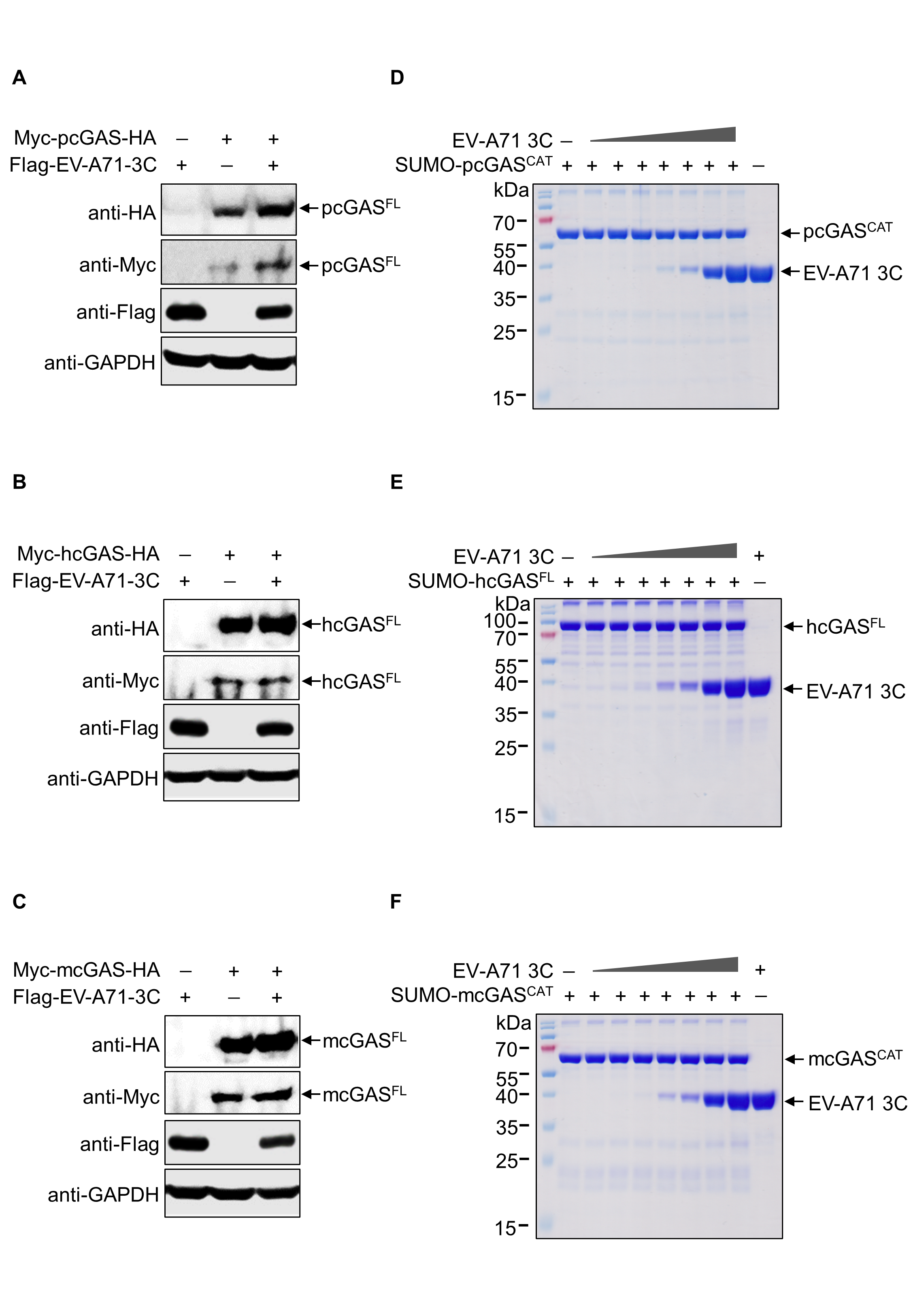

Supplement: S4 Fig — A-C, Western blot analysis of pcGAS, hcGAS or mcGAS cleavage and EV-A71 3C expression in HEK-293T cells transfected with 1.5 μg FLAG-EV-A71 3C plasmid and 2 μg wild-type Myc-pcGAS-HA (A), Myc-hcGAS-HA (B), or Myc-mcGAS-HA (C) plasmid for 24 h using anti-Myc, anti-HA and anti-Flag antibodies, respectively. D-F, SDS-PAGE analysis of in vitro cleavage of pcGAS, hcGAS or mcGAS in a 25-μl reaction containing 10 μg SUMO-pcGASCAT (D), SUMO-hcGASFL (E) or SUMO-mcGASCAT (F) recombinant protein with different amounts of purified recombinant protein EV-A71 3C (0.01, 0.05, 0.1, 0.5, 1, 5, 10 μg) for 2 h at 37°C. (TIF) [file ppat.1011641.s004.tif]

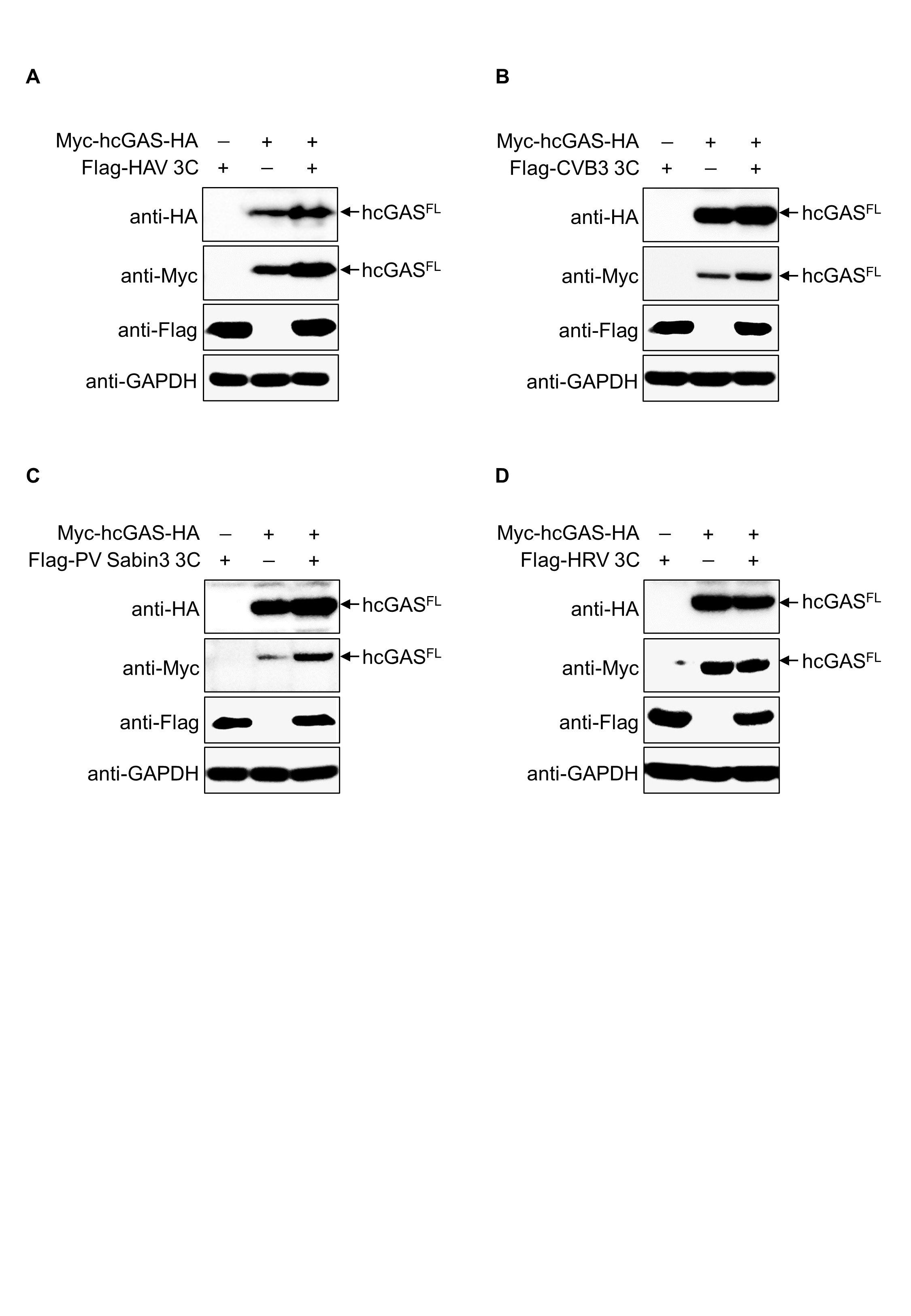

Supplement: S5 Fig — A-D, Western blot analysis of hcGAS cleavage and 3C expression in HEK-293T cells transfected with 2 μg wild-type Myc-hcGAS-HA plasmid and 1.5 μg FLAG-tagged plasmid encoding HAV 3C (A), 3C from CVB3-28 virus (B), 3C from Human poliovirus 3 strain Sabin 3 (C) or 3C from HRV-A16 virus (D) for 24 h using anti-Myc, anti-HA and anti-Flag antibodies. (TIF) [file ppat.1011641.s005.tif]

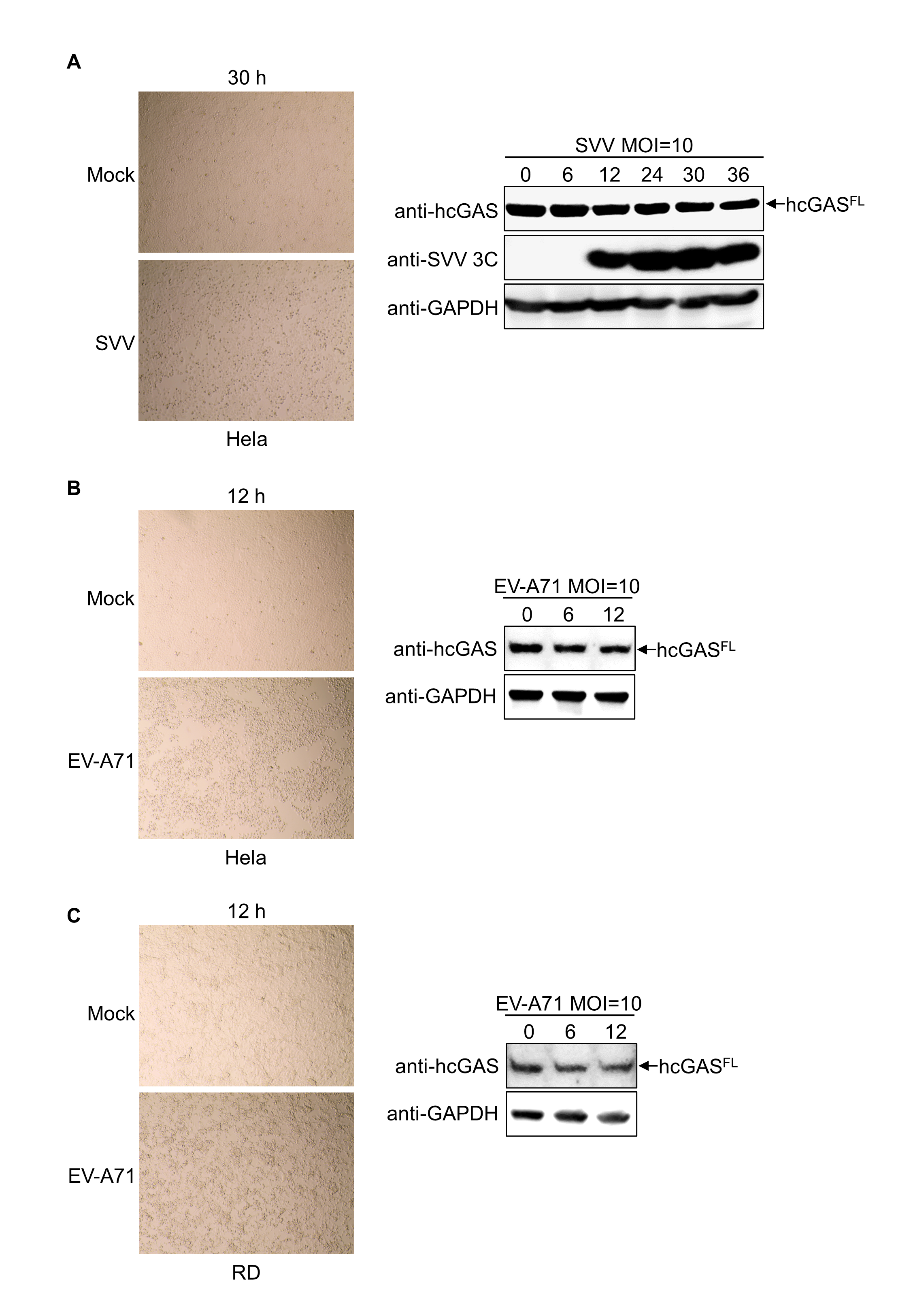

Supplement: S6 Fig — A, Hela cells were infected with SVV (MOI = 10) and harvested at 0, 6, 12, 24, 30 and 36 h post-infection. Cytopathic effect (CPE) in Hela cells without or with SVV infection for 30 h was shown by microscopy. The expression of endogenous hcGAS and SVV 3C was analyzed by Western blot. B, Hela cells were infected with EV-A71 (MOI = 10) and collected at 0, 6 and 12 h post-infection. Cytopathic effect (CPE) in Hela cells without or with EV-A71 infection for 12 h was shown by microscopy. The expression of endogenous hcGAS was detected by Western blot using anti-cGAS antibody. C, RD cells were infected with EV-A71 (MOI = 10) and collected at 0, 6 and 12 h post-infection. Cytopathic effect (CPE) in RD cells without or with EV-A71 infection for 12 h was shown by microscopy. The expression of endogenous hcGAS was analyzed by Western blot using anti-cGAS antibody. (TIF) [file ppat.1011641.s006.tif]

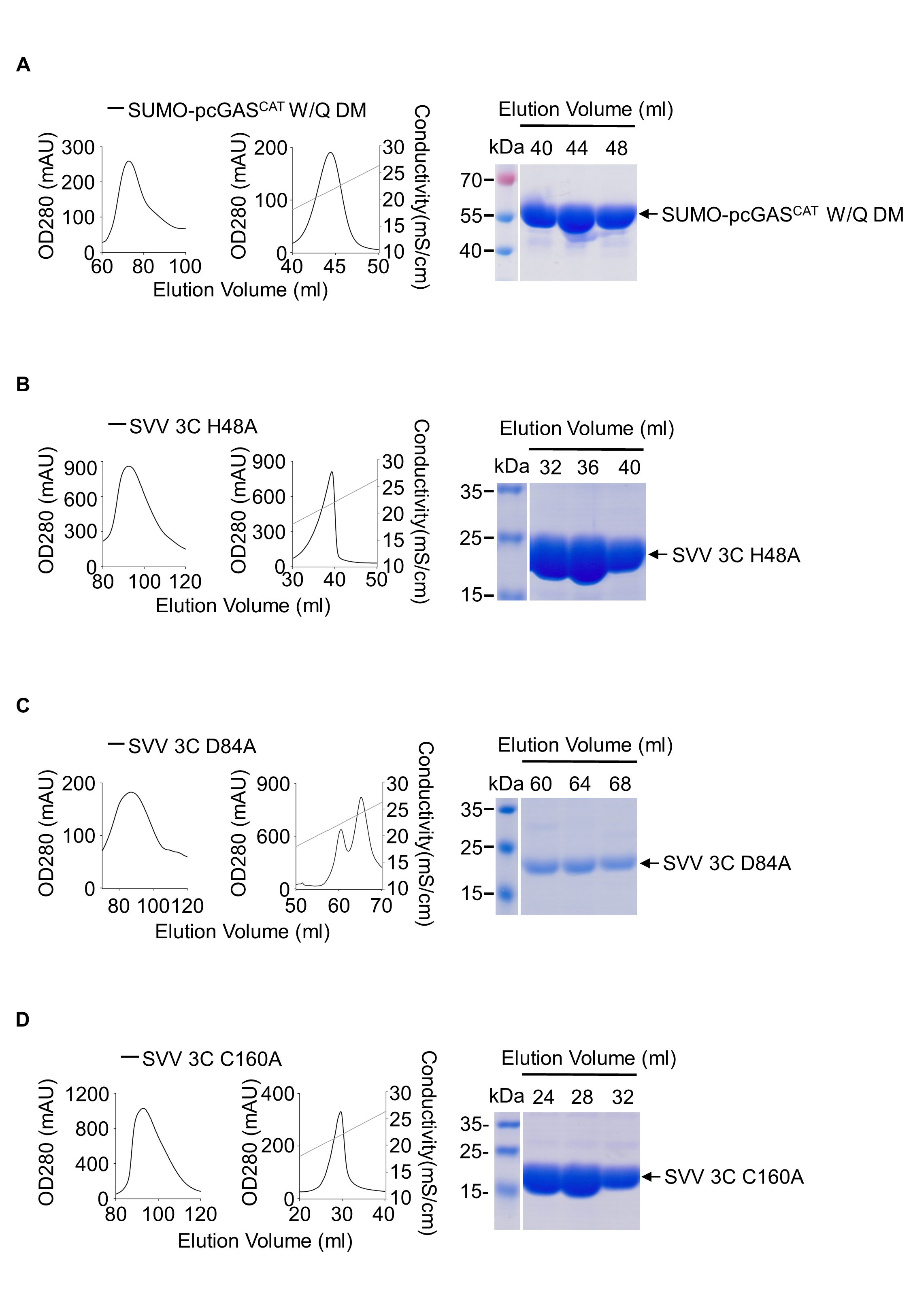

Supplement: S7 Fig — A, The SUMO-pcGASCAT mutant (W137A/Q140A) recombinant protein was purified by FPLC and ion exchange, followed by SDS-PAGE analysis. B-D, The SUMO-SVV 3C H48A (B), SUMO-SVV 3C D84A (C) and SUMO-SVV 3C C160A (D) mutant recombinant proteins were cleaved by SUMO protease overnight at 4°C. Then these mutant recombinant proteins were purified by FPLC and ion exchange, followed by SDS-PAGE analysis. (TIF) [file ppat.1011641.s007.tif]

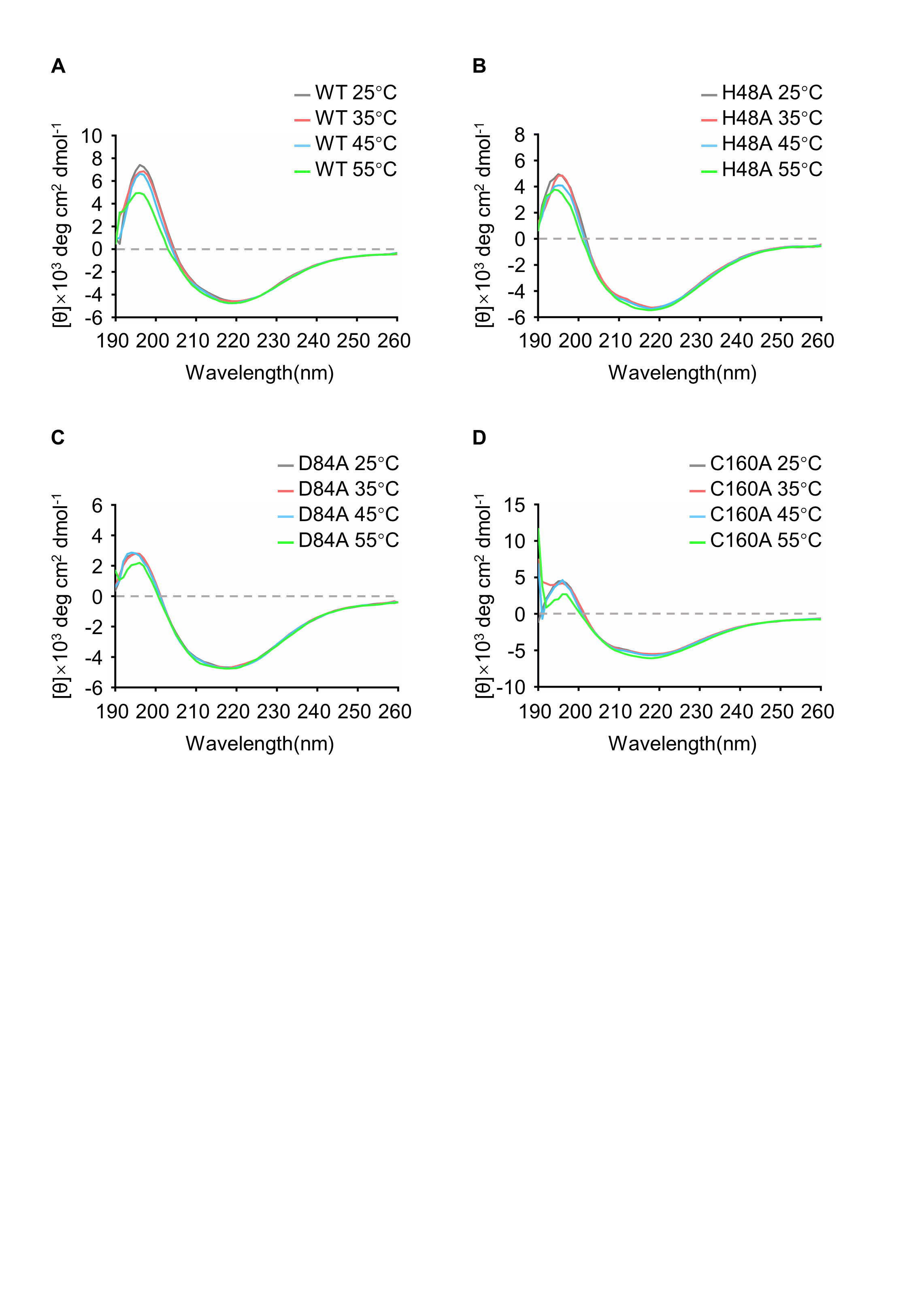

Supplement: S8 Fig — A-D, The purified SVV 3C protein and its mutants were diluted 100-fold with 10 mM Tris-HCl to obtain 0.2 mg/ml protein. Circular dichroism spectra (190 nm to 260 nm) of SVV 3C WT (A), 3C H48A (B), 3C D84A (C) and 3C C160A (D) proteins were tested at different temperatures (25°C, 35°C, 45°C and 55°C). (TIF) [file ppat.1011641.s008.tif]

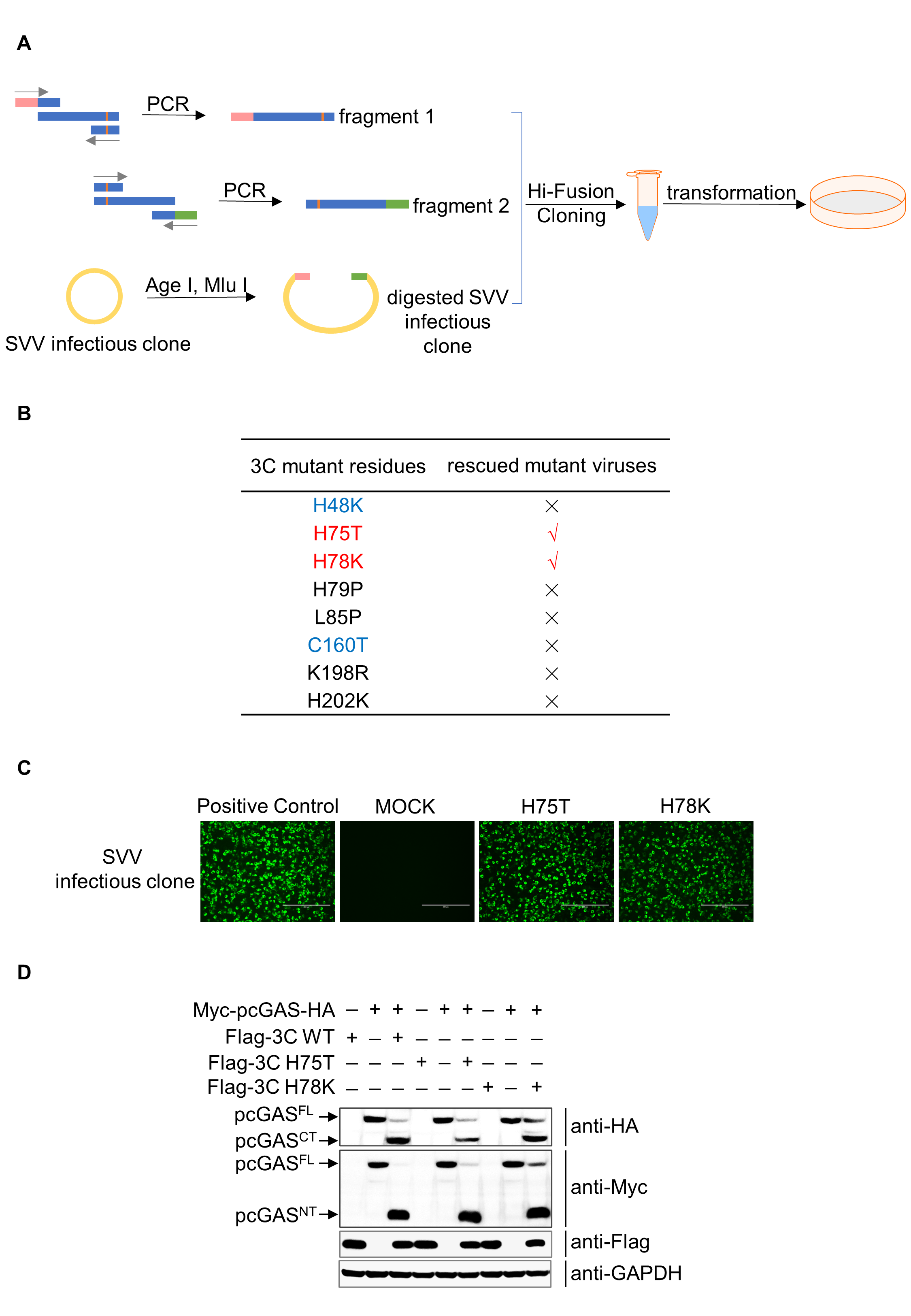

Supplement: S9 Fig — A, schematic diagram showing construction of plasmids with mutated 3C Protease. B, C, Rescue of 3C mutant virus. We tried to mutate the 3C residues listed in the table (B), and transfected SVV infectious clone with 3C mutant into cells (C). Tick means successfully rescuing the 3C mutant virus (B). Successfully rescued virus carried GFP fluorescence (C). D, Western blot analysis of pcGAS cleavage and SVV 3C expression in HEK-293T cells transfected with 2 μg wild-type pcGAS and 1.5 μg wild-type or mutant SVV 3C (3C WT, 3C H75T or 3C H78K) expression plasmid for 24 h using anti-Myc, anti-HA and anti-Flag antibodies. (TIF) [file ppat.1011641.s009.tif]
